# Association of Vegetarian and Vegan Diets with Cardiovascular Health: An Umbrella Review of Meta-Analysis of Observational Studies and Randomized Trials

**DOI:** 10.3390/nu15194103

**Published:** 2023-09-22

**Authors:** Honoria Ocagli, Giacomo Berti, Davide Rango, Federica Norbiato, Maria Vittoria Chiaruttini, Giulia Lorenzoni, Dario Gregori

**Affiliations:** Unit of Biostatistics, Epidemiology and Public Health, Department of Cardiac, Thoracic, Vascular Sciences, and Public Health, University of Padova, via Loredan 18, 35121 Padova, Italy; honoria.ocagli@unipd.com (H.O.); giacomo.berti@studenti.unipd.com (G.B.); davide.rango@studenti.unipd.com (D.R.); federica.norbiato@studenti.unipd.com (F.N.); mariavittoria.chiaruttini@studenti.unipd.com (M.V.C.); giulia.lorenzoni@unipd.com (G.L.)

**Keywords:** vegetarian diets, umbrella review, cardiovascular outcomes, cerebrovascular disease

## Abstract

Background: Cardiovascular diseases (CVDs) are leading global causes of mortality. Unhealthy diets have been linked to an increased risk of CVD, while plant-based diets have shown potential protective effects. This umbrella review summarizes the evidence on the association between vegetarian diets and cardiovascular and cerebrovascular diseases. Methods: PubMed, Scopus, Embase, CINAHL, Cochrane, and Web of Science were consulted. Meta-analyses grouped by author and outcome were performed. The heterogeneity was evaluated using I^2^ statistics. Results: There was a 41.2% risk reduction for cerebrovascular disease. CVD incidence had a 29% reduced risk. CVD mortality had a 13.8% risk reduction, while IHD incidence had a 24.1% reduction, but with high heterogeneity. IHD mortality showed a significant 32.1% risk reduction. Ischemic stroke had a significant 32.9% risk reduction across six studies. Stroke incidence showed a significant 39.1% risk reduction in a single study. There was a non-significant 11.6% risk reduction for stroke mortality with moderate heterogeneity. Conclusion: Healthier diets are associated with reduced risks of cerebrovascular disease, CVD incidence, IHD mortality, and ischemic stroke. However, evidence quality and consistency vary, emphasizing the need for more research. Policymakers and healthcare professionals should prioritize promoting healthy diets for CVD prevention.

## 1. Introduction

Cardiovascular diseases (CVDs), including coronary artery disease (CAD), heart failure, and cardiac death, significantly burden the global population. According to the World Health Organization (WHO), cardiovascular diseases are the leading cause of death globally, accounting for approximately 17.9 million deaths annually [1,2]. Among these, CAD, characterized by the narrowing or blockage of the coronary arteries, is the most common form of cardiovascular disease. CAD is a major contributor to heart attacks, angina, and other ischemic heart conditions. It affects individuals’ physical health and well-being and has profound socioeconomic implications [3]. The economic burden associated with CAD is immense, encompassing healthcare costs, lost productivity, and decreased quality of life [4].

Cerebrovascular diseases, such as strokes, significantly impact global health. Strokes occur when the blood supply to the brain is interrupted or reduced, leading to brain damage [5]. They can result in long-term disabilities, cognitive impairments, and even death. Strokes are a leading cause of adult disability and the second leading cause of death worldwide, accounting for approximately 6.2 million deaths yearly [6].

Given the substantial impact of cardiovascular and cerebrovascular diseases on individual health and global healthcare systems, there is a critical need to explore effective preventive measures and identify modifiable risk factors [7]. Unhealthy eating patterns, characterized by high intake of saturated fats, cholesterol, and processed foods, have been widely associated with an increased risk of CAD and cardiac death [8,9]. Conversely, diets rich in fruits, vegetables, whole grains, and lean proteins, such as The Dietary Approaches to Stop Hypertension (DASH), have shown potential protective effects against these conditions [10,11].

Furthermore, the rise in popularity of vegan and vegetarian diets has led to growing interest in exploring their potential benefits for primary and secondary prevention of cardiovascular health [12]. Recent studies have suggested an association between vegan and vegetarian diets and better cardiovascular health compared to no-plant-based diets. The study by Ivanova et al. [13] highlighted the potential of plant-based diets for weight control, emphasizing their positive effects on gut microbiota, insulin sensitivity, and other metabolic markers. Kahleova et al. [14] explored the cardio-metabolic benefits of plant-based diets, noting their association with decreased mortality and reduced risks of obesity, type 2 diabetes, and coronary heart disease. The study also suggested significant reductions in the risks of coronary heart disease and cerebral vascular disease events.

We propose an umbrella review of published meta-analyses to understand better the available evidence for the association between vegetarian diets and cardiovascular outcomes. Umbrella reviews of existing systematic reviews and/or meta-analyses, called overviews in the *Cochrane Handbook*, use systematic reviews (SRs) methodology on existing SRs or meta-analyses [15]. Other umbrella reviews in the existing body of research on this topic are available, comparing vegetarian and omnivorous diets and various health outcomes [16,17,18]. In the study of Oussalah et al. [17], compared to omnivorous diets, vegetarian diets showed lower blood cholesterol levels and a reduced risk of adverse health outcomes, including diabetes, heart disease, and cancer. Seventh-Day Adventist (SDA) vegetarians had an even lower risk than non-SDA vegetarians. However, vegetarian diets adversely affected one-carbon metabolism markers, like lower vitamin B12 and higher homocysteine levels. In the present umbrella review, we have focused on cardiovascular disease. Another umbrella review found that plant-based diets had significant effects on anthropometric parameters (e.g., body weight, BMI, waist circumference), but not cardiometabolic markers (e.g., diastolic blood pressure, HDL-C, triglyceride, LDL-C, and fasting blood glucose) in adults with Western eating habits [18]. Dinu et al. [16] summarized and evaluated the effects of different diets (e.g., low-carbohydrate, high-protein, low-fat, paleolithic, low-glycemic-index/load, intermittent energy restriction, Mediterranean, Nordic, vegetarian, Dietary Approaches to Stop Hypertension (DASH), and portfolio dietary pattern) on anthropometric parameters and cardiometabolic risk factors. A vegetarian diet showed weak evidence of an improvement in anthropometric parameters and cardiometabolic markers (e.g., total and LDL cholesterol, glucose, HbA1c, and blood pressure).

The present study aims to summarize the available evidence on the existing meta-analysis on the effect of vegetarian and vegan diets on cardiovascular and cerebrovascular disease risk.

## 2. Materials and Methods

The preferred reporting items for systematic reviews and meta-analyses (PRISMA) [19,20] guidelines were used to conduct this umbrella review. A PICO strategy [21] was formulated for Problem (P), vegetarian or vegan diets are associated with cardiovascular outcomes compared to other diets; Intervention (I), vegetarian or vegan diet or plant-based diet; Comparison (C), non-vegetarian diets; outcome (O), CVD events, coronary heart disease (CHD), CVD mortality, CHD mortality, and ischemic stroke.

### 2.1. Search Strategy and Information Source

The search strategy was first created for PubMed and then translated into the other database languages with the help of the tool Polyglot, https://sr-accelerator.com/#/polyglot (accessed on 15 July 2023) [22], enriching the string with terms from the database-specific thesaurus. The search was carried out on the title, abstract, and keywords in each database. The filter for the systematic review and meta-analysis was applied based on the filters available for systematic reviews, meta-analyses, health technology assessments, and indirect treatment comparisons https://searchfilters.cadth.ca/list?q=&ps=20&topic_facet=health%20technology%20assessments%20000000%7CHealth%20technology%20assessments&p=1 (accessed on 14 July 2023) [23,24,25,26]. The search strategy was based on the concepts of “vegetarian” or “vegan” or plant-based diet and the cardiovascular outcomes, such as “cardiovascular mortality”, “coronary heart disease”, and “stroke”. The detailed search strategy is available in the Supplementary Material Table S1.

PubMed, Embase (through Ovid), Scopus, CINAHL, Web of Science, and Cochrane Database were searched from inception to July 2022.

### 2.2. Eligibility Criteria

Studies were included according to the inclusion criteria enlisted in Table 1.

### 2.3. Selection Process

The web-based collaboration software platform Covidence was used (Covidence systematic review software, Veritas Health Innovation, Melbourne, Australia; Available at www.covidence.org) (accessed on 14 July 2023) to select the studies from title/abstract screening to the data extraction phase. The authors GB and DR independently reviewed the titles and abstracts of all the articles identified. GB and FN reviewed the full-text articles for eligibility. Both in title/abstract and full-text screening, disagreements were solved through discussion.

### 2.4. Data Extraction

GB and DR independently extracted data from eligible articles. Disagreement in data extraction was solved through discussion and, when needed, with a third reviewer’s support. The following information was collected through the Covidence platform: first author, year of publication, aim of the study, study design, number of patients, outcome, diet assessment method, effect size, 95% confidence intervals (CIs) risk of bias (RoB) tool and evaluation, number of cases and controls (in case-control studies), and number of events and population (in cross-sectional and prospective cohort studies). Outcomes were categorized as follows: Cardiovascular (CVD) events, CVD mortality, coronary heart disease (including ischemic heart disease), CHD mortality (including CHD mortality), and ischemic stroke. 

### 2.5. Quality Assessment

GB and DR independently evaluated the methodological quality of the studies included using the AMSTAR 2 Checklist [27]. The tool contains 16 questions and is not intended to generate an overall score. It contains critical and non-critical items. The studies were rated as high (no or one non-critical weakness), moderate (more than one non-critical weakness), low (one critical flaw, with or without non-critical weaknesses), or critically low quality (more than one critical flaw, with or without non-critical weaknesses), as suggested by the author. 

### 2.6. Statistical Analysis

The current study examines a single exposure factor with multiple outcomes of interest, and the analyses have been conducted at two levels of detail. First, the studies with the same outcome of interest were grouped within each meta-analysis. Second, the studies with the same outcome were grouped, regardless of their source meta-analysis. 

To conduct the meta-analysis, we gathered the following information for each study: (i) the risk measure used (either Risk Ratio—RR or Hazard Ratio—HR); (ii) the number of cases exposed (vegetarian); (iii) the number of cases non-exposed (non-vegetarian); (iv) the total number of exposed individuals; (v) the total number of non-exposed individuals; (vi) the estimate of RR or HR, along with their corresponding 95% Confidence Interval (95% CI); (vii) the Risk of Bias (RoB) tool; and (viii) the RoB score.

#### Non-Independence of Effect Sizes

Considering non-independence in the effect sizes involved three dependencies: hierarchical, multivariate, and partial [28,29]. Hierarchical dependence was identified when effect sizes were nested within a larger factor. An example occurs when multiple effect sizes come from independent studies reported in the same paper. Multivariate dependence was evident when effect sizes were derived from the same participants. For instance, this situation arises when several effect sizes are calculated from the same participants, who have completed multiple outcomes at a specific time-point or the same outcome at multiple time-points. Partial dependence was observed when effect sizes were derived from partly the same participants. This situation arises when several effect sizes in a meta-analysis originate from studies that compare independent experimental or exposed groups to a single control or non-exposed group. 

When hierarchical dependence was present in the data, a combined effect size across dependent studies was computed [28]. 

When multivariate dependence was present in the data, a combined effect size was computed across outcomes or time-points derived from the same units. More precisely, all dependent effect sizes derived from the same units are resumed to a unique effect size by estimating the non-weighted mean of all effect sizes [28]. The correlation among these effect sizes is used to calculate the variance of this combined effect size, as derived from the standard formula [28]. The sample size associated with this unique effect size equals the largest sample size that completed an outcome or time-point.

When partial dependence was present in the data, the shared group was split into several independent subgroups of smaller sample sizes, as described in the Cochrane Handbook [30]. More precisely, the number of participants in each independent subgroup is obtained by dividing the total number of participants in a shared group by the number of non-shared groups. These corrected sample sizes are used to re-estimate the effect sizes and their variance.

The summary effect size and its 95% CI were estimated using random effects models. In the case of effect sizes expressed by different measures, the Odds Ratio (OR) was used as the main effect size measure, and RR and HR were converted into an OR; moreover, to facilitate the comparison of effect sizes between meta-analyses, we reported the estimated effect sizes also in the equivalent odds ratio (eOR) by the methods implemented in the {metaumbrella} R package [31].

For the summary random effects, we estimated (only if the number of studies in the meta-analysis is equal or larger to 3) the 95% prediction interval (PI), which further accounts for the degree of between-study heterogeneity and gives a range for which we are 95% confident that the effect in a new study examining the same association lies within [32].

Statistical heterogeneity between studies was evaluated using the I^2^ statistic [33].

### 2.7. Credibility Assessment

To detect any evidence of biased study effects, we performed the Egger’s regression asymmetry test [34] and the standard error of the effect size (under random effects) for the largest study of each meta-analysis. The largest study was defined based on the smallest standard error.

Finally, we stratified the evidence related to the observed associations according to the criteria described in Fusar-Poli & Radua [35]. This classification proposes to stratify evidence into five ordinal classes: “Class I”, “Class II”, “Class III”, “Class IV”, and “Class ns”. (Supplementary Table S2).

## 3. Results

**Figure 1:**
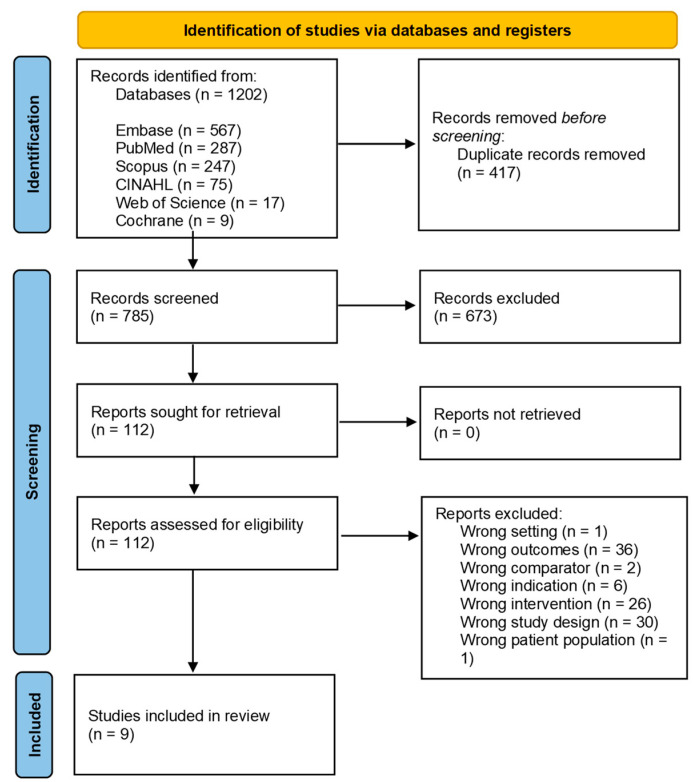
PRISMA Flow Diagram of the study selection process.

The search identified 1202 articles: 417 were removed as duplicates, and 673 were excluded from title/abstract screening. Of the 112 articles included in the full-text screening phase, 103 were excluded (Figure 1). Table S3 reports the list of excluded articles in full-text screening with reason of exclusion. Nine articles were included in the analysis. 

### 3.1. Study Characteristics

The nine systematic reviews assessed cardiovascular events [36,37,38], CVD mortality [38,39,40], coronary heart disease [36,37,39,41,42], CHD mortality [39,40,43], and ischemic stroke [37,38,39,44]. In Table S4 are reported the characteristics of the included meta-analysis in this umbrella review. The meta-analysis considered from 1 to 17 studies for each specific outcome. The number of participants varies between 44,561 [39] and 770,867 [37].

#### 3.1.1. Cardiovascular Events 

For what concerns cardiovascular events, Quek et al. [38] found that greater adherence to an overall plant-based dietary pattern was associated with a lower risk of CVD and CVD incidence, and healthful plant-based diets were associated with decreased CVD incidence. Also, Dybvik et al. [37] found that both vegetarians and vegans had a reduced risk of cardiovascular disease compared to non-vegetarians. However, no significant association was found for total cardiovascular events in the study of Dinu et al. [36]. 

#### 3.1.2. CVD Mortality

Quek [38] determined unhealthful plant-based diets were linked to increased cardiovascular mortality. Kwok et al. [42] examined the association between a vegetarian diet and cardiovascular mortality among Seventh-Day Adventists (SDA) and other cohorts. They found that SDA studies showed a greater effect size, with a lower risk of death and ischemic heart disease among vegetarians, but the effect was less clear in non-SDA studies. Similar results were reported by Glenn et al. [39].

#### 3.1.3. Coronary Heart Disease and CHD Mortality

Glenn et al. [39] found that a vegetarian dietary pattern was associated with reduced coronary heart disease mortality and incidence. 

Dinu et al. [36], Dybvik et al. [37], Jafari et al. [40], Huang et al. [41], Kwok et al. [42], and Jabri et al. [43] found that vegetarians and vegans had a lower risk of incidence and mortality from ischemic heart disease.

#### 3.1.4. Ischemic Stroke

Quek et al. [38], Glenn et al. [39], and Jabri et al. [43] found no significant difference in stroke risk compared to meat-eaters. Also, Lu et al. [44] found no significant association between adhering to vegetarian diets and the overall risk of incident stroke. However, subgroup analyses suggested that studies conducted in Asia and those with participants aged 50–65 showed a lower risk of stroke in vegetarians.

Conversely, Dybvik et al. [37] found that both vegetarians and vegans had a reduced risk of stroke compared to non-vegetarians. Similar results were reported by Huang et al. [41]. Vegetarians also had a 16% lower mortality from circulatory diseases and a 12% lower mortality from cerebrovascular disease. 

### 3.2. Definition of Vegetarian

Vegetarian and vegan dietary patterns have different characteristics in relation to food selection and reasons that motivate the adoption of such patterns [33]. The studies considered the intervention differently: vegetarian status was defined as excluding meat, poultry, fish, and seafood [44]. Some studies included lacto-ovo vegetarians (who did not eat meat or fish or ate these foods less than once a week, but did eat eggs, or dairy products, or both) and vegans altogether (who did not eat meat, fish, eggs, or dairy products) [36,37,41] or vegetarians and Seventh-Day Adventists altogether. The study of Jabry et al. [43] included a vegetarian diet (defined as one excluding meat, pesco) or a semi-vegetarian diet (defined as a meal containing little animal and predominantly plant-based protein). Others considered plant-based dietary patterns as higher consumption of plant-based foods and lower consumption or exclusion of animal-based foods.

Among the studies considered in the meta-analysis included were reported 14 prospective cohorts: Adventist Health study 1, Adventist Mortality study in the meta-analysis of Jabri et al. [43]; Adventist Health Study in Kwok et al. [42], the Adventist Health study 2 in Glenn et al. [39], Jafari et al. [40], and Kwok et al. [42]; Tzu Chi Health Study, Tzu Chi Vegetarian Study, Nurses’ Health Study I and II, and Health Professionals Follow-Up Study in Lu et al. [44]; EPIC Oxford study in Dinu et al. [36], Glenn et al. [39], Kwok et al. [42], and Jabri et al. [43]; German Vegetarian study in Dinu et al. [36], Dybvick et al., Glenn et al. [39], Huang et al. [41], Jabri et al. [43], Jafari et al., and Kwok et al. [42]; Health Food Shoppers Study in Glenn et al. [39] and Kwok et al. [42]; Japanese Zen Priest study in Jabri et al. [43] and Kwok et al. [42]; National Health and Nutrition Examination Survey in Jafari et al. [40]; and Netherlands Adventist study in Huang et al. [41], Jabri et al. [43], and Kwok et al. [42].

### 3.3. Meta-Analysis Results Grouping by Author and Outcome

Table 2 presents a summary of included studies on the association between dietary factors and CVD outcomes. Each row represents a different study, and the columns provide information on the year of publication, the specific CVD outcome studied, the number of studies included in the meta-analysis, the total number of individuals, number of cases and controls, and the effect size with its corresponding 95% confidence interval (CI). The effect sizes are presented as relative risks (RRs) or hazard ratios (HRs), depending on the study outcome. The estimate of the umbrella review is presented as equivalent odds ratios (ORs). Additionally, the table includes information on the heterogeneity (I^2^) of the results, the classification of the study as proposed by Fusar-Poli & Radua [35] (Class), and the RoB (%). There is only one study that considers only one outcome [44]; all the others consider multiple outcomes. 

Dinu et al. [36] analyzed four studies for CVD mortality with a total of 107,285 participants (3778 cases and 103,507 controls). The combined effect size (eOR) was 0.847, indicating a non-significant 15.3% reduction in CVD mortality risk for those following healthy dietary patterns. For IHD mortality, they analyzed six studies with 225,618 participants (5169 cases and 220,449 controls). The eOR was 0.839, showing a non-significant 16.1% reduction in IHD mortality risk.

Dybvik [37] investigated various CVD outcomes. For Cerebrovascular disease, they analyzed three studies with 121,850 participants (1096 cases and 120,754 controls). The eOR was 0.588, indicating a significant 41.2% reduction in Cerebrovascular disease risk among those with healthy dietary patterns. They analyzed one study for CVD incidence with 398,448 participants (106,690 cases and 291,758 controls). The eOR was 0.71, showing a significant 29% reduction in CVD incidence risk.

Jabri et al. [43] studied CVD, IHD, and Stroke mortality. They analyzed one study for CVD mortality with 47,254 participants (950 cases and 46,304 controls). The eOR was 0.543, indicating a significant 45.7% reduction in CVD mortality risk. For IHD mortality, they analyzed four studies with 70,942 participants (1342 cases and 69,600 controls). The eOR was 0.54, showing a significant 46% reduction in IHD mortality risk.

Jafari et al. [40] investigated CVD, IHD, and Stroke mortality. For CVD mortality, they analyzed five studies with 101,665 participants (14,091 cases and 87,574 controls). The eOR was 0.827, indicating a non-significant 17.3% reduction in CVD mortality risk. For IHD mortality, they analyzed eight studies with 313,305 participants (2912 cases and 310,393 controls). The eOR was 0.629, showing a significant 37.1% reduction in IHD mortality risk.

For Ischemic Stroke, Lu et al. [44] analyzed four studies with 679,034 participants (12,791 cases and 666,248 controls). The eOR was 0.688, indicating a significant 37.5% reduction in Ischemic Stroke risk.

Quek et al. [38] investigated CVD mortality and Ischemic Stroke outcomes. For CVD mortality, they analyzed one study with 4282 participants (1565 cases and 2717 controls). The eOR was 0.681, indicating a significant 31.9% reduction in CVD mortality risk. For Ischemic Stroke, they analyzed two studies with 61,540 participants (604 cases and 60,936 controls). The eOR was 0.473, showing a significant 52.7% reduction in Ischemic Stroke risk.

### 3.4. Meta-Analysis Results Grouping by Outcome

**Figure 2:**
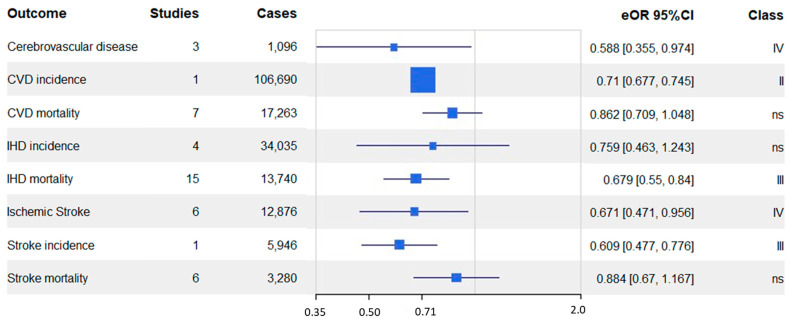
Forest plot of all non-overlapping meta-analyses of included studies.

Table 3 and Figure 2 report the meta-analysis results grouped by the outcome. In Table S6, the credibility assessment of the results grouped by the outcome of the meta-analysis included in the umbrella review is reported. Grouping according to outcome, the studies resulted as follows: cerebrovascular disease (*n* = 3), CVD incidence (*n* = 1), CVD mortality (*n* = 7), IHD incidence (*n* = 4), IHD mortality (*n* = 15), stroke incidence (*n* = 1), stroke mortality (*n* = 6). 

For cerebrovascular disease, the pooled analysis of three studies showed a significant 41.2% reduction in risk (eOR 0.588, 95% CI 0.36–0.97) among individuals with healthier dietary patterns. Similarly, one study on CVD incidence demonstrated a 29% reduced risk (eOR 0.71, 95% CI 0.68–0.75) associated with healthier diets.

The association between dietary patterns and CVD mortality showed some heterogeneity, with a 13.8% reduction in risk (eOR = 0.862, 95% CI 0.71–1.05). On the other hand, the relationship between dietary patterns and IHD incidence was less conclusive, with a 24.1% reduction in risk (eOR 0.759, 95% CI 0.46–1.24), but with high heterogeneity across the four included studies.

Regarding IHD mortality, the meta-analysis of 15 studies showed a significant 32.1% reduction in risk (eOR 0.679, 95% CI 0.55–0.84) associated with healthier dietary patterns, though the results had moderate heterogeneity.

Ischemic stroke demonstrated a significant 32.9% reduction in risk (eOR 0.671, 95% CI 0.47–0.96) across six studies, but again, there was notable heterogeneity in the findings.

For stroke incidence, a single study indicated a significant 39.1% risk reduction (eOR 0.609, 95% CI 0.48–0.78) with healthier diets, but this finding should be interpreted with caution due to the limited number of studies.

Lastly, for stroke mortality, the meta-analysis of six studies showed a non-significant 11.6% reduction in risk (eOR 0.884, 95% CI 0.67–1.17), and moderate heterogeneity was observed in the results.

In Figure 2 is depicted the forest plot with the summary effect in terms of eOR with the 95% CI for each cardiovascular outcome evaluated in this umbrella review. 

A vegetarian diet is associated with a reduced risk of all cardiovascular outcomes, especially for CVD incidence (eOR 0.7, 95% CI 0.68–0.75), IHD mortality (eOR 0.7, 95% CI 0.55–0.84), MI incidence (eOR 0.6, 95% CI 0.45–0.72), stroke incidence (eOR 0.6, 95% CI 0.48–0.78), cerebrovascular disease (eOR 0.6, 95% CI 0.36–0.97), and ischemic stroke (eOR 0.7, 95% CI 0.47–0.96). Instead, for CVD mortality (eOR 0.9, 95% CI 0.71–1.05), stroke mortality (eOR 0.9, 95% CI 0.67–1.17), and IHD incidence (eOR 0.8, 95% CI 0.47–1.25), the vegetarian diet is less protective (Figure 2). 

### 3.5. Evaluation of Bias, Heterogeneity, and Quality

Table S5 and Table S6 report the credibility assessment of the results, grouped by author and outcome and by outcome, respectively, of the meta-analysis included in the umbrella review. 

According to the AMSTAR-2 assessment, one meta-analysis was considered of high quality [37], two of moderate quality [25,32], one of low quality [36], and the remaining of critically low quality [6,37,38,39,40,41,42,43,44] (Table S7). All the meta-analyses provided the research question and PICOs components (Q1), used a comprehensive literature search strategy (Q4), performed data extraction in duplicate (Q6), and reported any potential source of conflict of interest (Q16). Reporting of sources of findings (Q10) was missing in seven meta-analyses; (Q13) in six; and detailed description of included studies (Q8), satisfactory techniques to assess ROB (Q9), and evaluation of RoB’s impact on meta-analysis results (Q12) in five studies (Table S7). 

### 3.6. Strength of Evidence

The I^2^ represents the degree of heterogeneity across studies, where higher values indicate greater variability among study results; it varies between 50.19% and 96.30%, grouping by outcome in each meta-analysis (Table 2).

**Figure 3:**
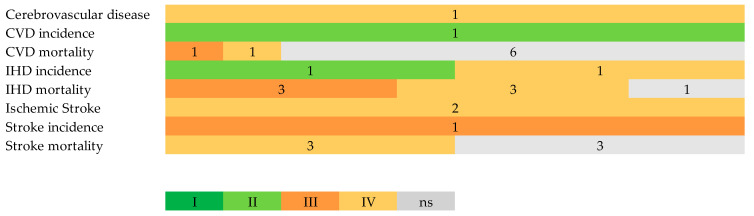
Summary of the strength of evidence for the evaluated health outcomes. Evidence class: Class I, convincing; Class II, highly suggestive; Class III, suggestive; Class IV, weak; NS, no evidence. The number reported refers to the number of studies considered.

Figure 3 shows the strength of the evidence according to the criteria level of significance for the random effect calculations, the sample size, the heterogeneity, the 95% CI, and the small study effects presence. Table S5 reports the credibility assessment of the results grouping by author and outcome of the meta-analysis included. The numbers refer to the number of meta-analyses with the level convincing (Class I), highly suggestive (Class II), suggestive (Class III), weak (Class IV), and no evidence (Class ns). No convincing level was found in the outcomes considered; the majority of studies have the non- significant class. Cerebrovascular disease was in class IV [37]; CVD incidence was in class II [37]; CVD mortality contained meta-analysis with no evidence [36,37,39,40,41,42] and classes III [38] and IV [43]; IHD incidence was in classes IV [37] and II [39]; IHD mortality was in no evidence [36], III [40,41,43], and IV [37,39,42]; ischemic stroke was in no evidence [44] and class IV [38,44]; MI incidence was in class III [37]; stroke incidence was in class III [37]; and stroke mortality was in the no evidence class [39,42,43] and class IV [37,40,41] (Table 2).

## 4. Discussion

The present umbrella review evaluates the association between vegetarian or vegan diets and cardiovascular events, CVD mortality, coronary heart disease, CHD mortality, and ischemic stroke.

The main results of the presented meta-analysis highlight the potential benefits of adopting plant-based dietary patterns in reducing the risk of certain cardiovascular diseases. The findings indicate a significant association between healthier diets and decreased risk of cerebrovascular disease and CVD incidence, with 41.2% (eOR 0.588, 95% CI 0.36–0.97) and 29% (eOR 0.71, 95% CI 0.68–0.75) risk reductions, respectively. Our findings align with those of Oussalah et al. [17] concerning heart disease. 

However, it is essential to note that the association between dietary patterns and CVD mortality exhibited some heterogeneity, with a 13.8% (eOR 0.86, 95% CI 0.71–1.05) risk reduction. This variability in results might be influenced by factors such as differences in study populations, dietary assessment methods, and other confounding variables not accounted for in the meta-analysis.

Furthermore, the relationship between dietary patterns and IHD incidence could have been clearer-cut, with a 24.1% (eOR 0.76, 95% CI 0.46–1.24) risk reduction and high heterogeneity across the included studies. This discrepancy highlights the need for more consistent and rigorous research to understand better the impact of dietary choices on the incidence of ischemic heart disease.

On the positive side, the meta-analysis of IHD mortality showed a significant 32.1% (eOR 0.679, 95% CI 0.55–0.84) risk reduction associated with healthier dietary patterns, indicating that conscious dietary choices could favor heart-healthy outcomes.

While the meta-analysis demonstrated a significant 32.9% (eOR 0.67,95% CI 0.55–0.84) reduction in the risk of ischemic stroke associated with healthier diets, the limited number of studies in the stroke incidence analysis warrants cautious interpretation. More studies are needed to validate this finding and to establish more substantial evidence for the preventive effects of dietary patterns on stroke incidence.

The results of the methodologic quality of the meta-analysis included in this study suggest that the current meta-analysis still needs to achieve a medium-to-high-quality score. According to the Eggers *p*-value, the publication bias was not significant for all outcomes of interest. The measure derives from the Eggers test, a statistical method used to assess the presence of publication bias or small study effects by examining the relationship between study effect sizes and their precision (inverse of standard error). None of the studies are in Class I. The quality of meta-analysis relies on the quality of included studies, so the results derived in this umbrella should be interpreted cautiously, especially for outcomes with few studies and of poor quality. All the included SRs evaluate the RoB with tools considered appropriate in the field of nutrition [45], except Huang et al. [41]. Moreover, Glenn et al. [39], Jabri et al. [43], and Jafari et al. [40] also use the Grading of Recommendations Assessment, Development, and Evaluation (GRADE) approach and Lu et al. the NutriGrade.

### Limitations

The results of this umbrella review should be interpreted cautiously, considering it suffers from several biases. 

First, most studies related to nutrition use a nonrandomized observational study design, which brings difficulties in detecting small effect sizes, accounting for confounding variables, inaccurate diet measurements, and reporting bias [46]. To solve these issues, appropriate study designs are needed, such as randomized trials [45,46,47]. Few primary studies achieved a high-quality score when evaluating the risk of bias. 

Moreover, individuals who opt for plant-based diets might be more health-conscious and informed about nutrition. However, a plant-based diet does not mean a healthy one, since some foods are classified as ultra-processed foods (UPFs) [48]. This could impact the outcomes of observational studies and ours. 

Another limit relies on the variability in diet definitions, populations, and duration of interventions. The studies considered vegetarians and vegans altogether or considered high intakes of vegetables. The definitions of the outcomes could vary according to the different studies. These differences provide estimates with wider confidence intervals, which strengthens the robustness of the protective effect. These variations introduce a certain level of uncertainty or “variability” into the results of the studies. However, rather than weakening the findings, this increased variability can strengthen the results’ significance. When the individual studies being analyzed in a meta-analysis have diverse methodologies, definitions, and outcomes, it can create a broader range of possibilities regarding the observed effects. So, this increased variability reinforces the significance of the findings. A wider confidence interval suggests more uncertainty about the true effect, but it also means that the effect could be larger or smaller than initially estimated. This uncertainty prompts researchers to be cautious and encourages them to consider a range of scenarios.

In the included meta-analysis, the same study cohorts were often considered; this is challenging in terms of exposure. In the analysis, we have taken into account three types of dependency effect sizes: hierarchical, multivariate, and partial. 

Other limitations are related to the nature of the umbrella review. In the realm of scientific inquiry, umbrella reviews are a powerful tool for synthesizing existing research findings. However, one must acknowledge that a notable challenge resides intrinsically within this methodology. This challenge pertains to the inherent complexity of integrating diverse studies and their outcomes into a cohesive narrative. In particular, the quality of our results relies on the reporting of the included studies [35]. Addressing all potential confounding variables reported in the original studies is challenging. This limitation can be circumvented with an individual-patient data analysis, which is not the aim of this study. Moreover, the generalizability of the findings is limited. While these results may be applicable to Western countries, given the predominant focus on nations like the USA, UK, and various European countries, including Germany, Spain, and the Netherlands, it is essential to tread cautiously. The inclusion of other countries, such as Japan, Taiwan, and Australia, in only a limited number of original studies can potentially reduce the generalizability. Lastly, the results contribute to the challenge of ecological fallacy. The risk lies in assuming that the aggregated patterns observed at the country level can directly translate to individual-level phenomena, which might not hold true for every case. Therefore, while the data provide insights into trends across these nations, their direct application to the behavior and characteristics of individuals within each country should be approached with consideration for the ecological fallacy and the potential nuances it entails. 

## 5. Conclusions

This umbrella review provides a comprehensive analysis of the published meta-analyses in relation to vegetarian diets and cardiovascular disease. Overall, these findings highlight the importance of healthy dietary patterns in preventing certain CVD outcomes. These findings should be interpreted with the emphasis that dietary recommendations for preventing cardiovascular disease (CVD) should shift towards promoting healthy dietary patterns, rather than solely focusing on individual food groups or nutrients [49].

In summary, when grouped by outcome, the results suggest that adopting healthier dietary patterns may be associated with reduced risks of cerebrovascular disease, CVD incidence, IHD mortality, and ischemic stroke. However, the evidence’s quality and the consistency of the findings varied across different outcomes, with some results showing significant reductions in risk and others needing to be more conclusive and non-significant. Further research and high-quality studies are required in order to better understand the role of dietary patterns in preventing different cardiovascular diseases.

Policymakers and healthcare professionals can utilize these findings to emphasize the importance of promoting healthy dietary habits in cardiovascular disease prevention initiatives and public health campaigns.

## Figures and Tables

**Table 1:** Eligibility criteria used in the present umbrella review.

Parameter	Inclusion	Exclusion
Population	Adults with age ≥ 16 years	Specific population (e.g., diabetic)
Intervention	Vegetarian diet, vegan diet, plant-based diet	An omnivorous diet with additional vegetable/fruit
Comparison	Non-vegetarian diet	
Outcome	Cardiovascular (CVD) events, CVD mortality, coronary heart disease (CHD), CHD mortality, ischemic stroke	Hemorrhagic stroke
Study design	Systematic reviews, including meta-analyses (Quantitative analysis) of prospective observational studies (cross-sectional, case-control, cohort) or randomized clinical trials (RCTs)	Systematic review without meta-analysis, narrative review

**Table 2:** Characteristics and quantitative synthesis of meta-analyses.

Author Year	Country	Outcome	N Studies	N Participants	Cases	Controls	Effect Size	eOR	eOR 95% CI	I^2^	Class *	RoB %
Dinu 2016 [36]	USA, Germany, UK	CVD mortality	4	107,285	3778	103,507	OR	0.85	0.61, 1.17	93.205	ns	44.05
		IHD mortality	6	225,618	5169	220,449	OR	0.84	0.62, 1.14	95.475	ns	40.69
Dybvik 2023 [37]	USA, Germany, UK, Taiwan	Cerebrovascular disease	3	121,850	1096	120,754	RR	0.588	0.36, 0.97	86.36	IV	0.00
		CVD incidence	1	398,448	106,690	291,758	RR	0.71	0.68, 0.75	1 study	II	0.00
		CVD mortality	1	36,346	987	35,359	HR	0.87	0.75, 1.01	1 study	ns	0.00
		IHD incidence	3	919,768	32,800	886,968	RR	0.605	0.44, 0.83	88.903	IV	0.00
		IHD mortality	7	266,473	8523	257,950	OR	0.733	0.59, 0.92	96.286	IV	0.00
		Stroke incidence	1	422,102	5946	416,156	RR	0.609	0.48, 0.78	1 study	III	0.00
		Stroke mortality	3	98,072	1417	96,655	RR	1.136	1.02, 1.26	0	IV	0.00
Glenn 2019 [39]	USA, Germany, UK	CVD mortality	4	107,285	3778	103,507	OR	0.846	0.61, 1.17	93.123	ns	55.95
		IHD incidence	1	44,561	1235	43,326	RR	1.474	1.32, 1.65	1 study	II	0.00
		IHD mortality	5	181,057	3934	177,123	OR	0.738	0.59, 0.92	80.08	IV	73.90
		Stroke mortality	4	145,326	1682	143,644	RR	0.995	0.75, 1.32	79.157	ns	67.48
Huang 2012 [41]	USA, Netherlands, UK, Germany, Japan	CVD mortality	2	49,158	1205	47,953	RR	0.792	0.37, 1.68	96.297	ns	100.00
		IHD mortality	6	92,314	2290	90,024	RR	0.63	0.49, 0.80	82.323	III	100.00
		Stroke mortality	5	80,993	1508	79,485	RR	0.734	0.56, 0.97	82.995	IV	100.00
Jabri 2021 [43]	USA, Netherlands, UK, Germany, Japan	CVD mortality	1	47,254	950	46,304	RR	0.543	0.47, 0.63	1 study	IV	0.00
		IHD mortality	4	70,942	1342	69,600	RR	0.54	0.41, 0.70	60.845	III	0.00
		Stroke mortality	3	60,103	1042	59,061	RR	0.722	0.46, 1.15	84.096	ns	0.00
Jafari 2022 [40]	USA, UK, Germany, Europe, Spain, Australia	CVD mortality	5	101,665	14,091	87,574	OR	0.827	0.63, 1.09	95.208	ns	35.75
		IHD mortality	8	313,305	2912	310,393	OR	0.629	0.48, 0.82	89.477	III	31.08
		Stroke mortality	3	68,144	731	67,413	RR	0.727	0.57, 0.93	50.186	IV	0.00
Kwok 2014 [42]	USA, Netherlands, UK, Germany, Japan	CVD mortality	6	156,443	4983	151,460	OR	0.827	0.62, 1.10	93.59	ns	62.84
		IHD mortality	11	270,678	6985	263,693	OR	0.753	0.61, 0.94	93.969	IV	35.33
		Stroke mortality	6	207,912	2721	205,191	RR	0.804	0.62, 1.05	96.291	ns	45.46
Lu 2021 [44]	USA, UK, Taiwan	Ischemic Stroke	6	679,034	12,791	666,243	RR	0.688	0.50, 0.95	73.083	IV	7.1 866
Quek 2021 [38]	Europe, North America, Asia, Europe	CVD mortality	1	4282	1565	2717	HR	0.681	0.58, 0.80	1 study	III	0.00
		Ischemic Stroke	2	61,540	604	60,936	RR	0.473	0.28, 0.79	61.765	IV	0.00

Abbreviations: CI, confidence interval; CVD, cardiovascular disease; eOR, equivalent odds ratio; IHD, ischemic heart disease; MI, myocardial infarction; RoB, risk of bias. * Evidence class: Class I, convincing; Class II, highly suggestive; Class III, suggestive; Class IV, weak; ns, non-significant.

**Table 3:** Characteristics and quantitative synthesis of meta-analyses.

Outcome	N Studies	Total N	Cases	Controls	Measure	eOR	eOR 95% CI	I^2^	Class	RoB %
Cerebrovascular disease	3	121,850	1096	120,754	RR	0.588	0.36, 0.97	86.36	IV	0
CVD incidence	1	398,448	106,690	291,758	RR	0.71	0.68, 0.75	1 study	II	0
CVD mortality	7	145,227	17,263	127,964	OR	0.862	0.71, 1.05	92.83	ns	16.31
IHD incidence	4	964,329	34,035	930,294	RR	0.759	0.46, 1.24	98.24	ns	0
IHD mortality	15	619,430	13,740	605,690	OR	0.679	0.55, 0.84	96.68	III	18.49
Ischemic stroke	6	692,386	12,876	679,510	RR	0.671	0.47, 0.96	78.19	IV	0
Stroke incidence	1	422,102	5946	416,156	RR	0.609	0.48, 0.78	1 study	III	0
Stroke mortality	6	196,925	3280	193,645	RR	0.884	0.67, 1.17	97.13	ns	40.97

Abbreviations: CI, confidence interval; CVD, cardiovascular disease; eOR, equivalent odds ratio; IHD, ischemic heart disease; RoB, risk of bias.

## Data Availability

The data presented in this study are available on request from the corresponding author. The data are not publicly available due to privacy restrictions.

## Supplementary Materials

The following supporting information can be downloaded at: https://www.mdpi.com/article/10.3390/nu15194103/s1, Table S1: Search strategies; Table S2: Credibility assessment of the results; Table S3: List of excluded articles in full-text screening with reason of exclusion; Table S4: Characteristics of included systematic reviews and meta-analyses; Table S5: Credibility assessment of the results grouping by author and outcome of the meta-analysis included in the umbrella review; Table S6: Credibility assessment of the results grouping by outcome of the meta-analysis included in the umbrella review; Table S7: AMSTAR-2 scoring of the meta-analysis included in the umbrella systematic review; S8: References.
