# Supplementary material for: Association of Vegetarian and Vegan Diets with Cardiovascular Health: An Umbrella Review of Meta-Analysis of Observational Studies and Randomized Trials
Source: Nutrients. 2023 Sep 22;15(19):4103. doi: 10.3390/nu15194103 (PMC10574056; doi:10.3390/nu15194103)
Supplement: Supplementary file 1 [file nutrients-15-04103-s001.zip › nutrients-2561478-supplementary.pdf]

Supplementary material

Table S1 Search strategies.

The filter for systematic review and meta-analysis was applied based on the filters available for systematic reviews, meta-analyses, health technology assessments and indirect treatment comparisons (https://searchfilters.cadth.ca/list?q=&ps=20&topic_facet=health%20technology%20assessments%20000000%7CHealth%20technology%20assessments&p=1) [1–4].

PubMed	("myocardial ischemia"[MeSH Terms] OR "myocardial ischaemia"[Title/Abstract] OR "ischemia myocardial"[Title/Abstract] OR "ischemias myocardial"[Title/Abstract] OR "Myocardial Ischemias"[Title/Abstract] OR "Ischemic Heart Disease"[Title/Abstract] OR "heart disease ischemic"[Title/Abstract] OR "disease ischemic heart"[Title/Abstract] OR "diseases ischemic heart"[Title/Abstract] OR "heart diseases ischemic"[Title/Abstract] OR "Ischemic Heart Diseases"[Title/Abstract] OR "coronary disease"[MeSH Terms] OR "coronary disease"[Title/Abstract] OR "Coronary Diseases"[Title/Abstract] OR "disease coronary"[Title/Abstract] OR "diseases coronary"[Title/Abstract] OR "Coronary Heart Disease"[Title/Abstract] OR "Coronary Heart Diseases"[Title/Abstract] OR "disease coronary heart"[Title/Abstract] OR "diseases coronary heart"[Title/Abstract] OR "heart disease coronary"[Title/Abstract] OR "heart diseases coronary"[Title/Abstract] OR "coronary artery disease"[MeSH Terms] OR "coronary artery disease"[Title/Abstract] OR "artery disease coronary"[Title/Abstract] OR "artery diseases coronary"[Title/Abstract] OR "Coronary Artery Diseases"[Title/Abstract] OR "Left Main Coronary Artery Disease"[Title/Abstract] OR "Left Main Disease"[Title/Abstract] OR "Left Main Diseases"[Title/Abstract] OR "Left Main Coronary Disease"[Title/Abstract] OR "Coronary Arteriosclerosis"[Title/Abstract] OR "atherosclerosis coronary"[Title/Abstract] OR "Coronary Atherosclerosis"[Title/Abstract] OR "arteriosclerosis coronary"[Title/Abstract] OR "Coronary Heart Disease"[Title/Abstract] OR "Myocardial Infarction"[MeSH Terms] OR "Myocardial Infarction"[Title/Abstract] OR "infarction myocardial"[Title/Abstract] OR "infarctions myocardial"[Title/Abstract] OR "Myocardial Infarctions"[Title/Abstract] OR "Cardiovascular Stroke"[Title/Abstract] OR "stroke cardiovascular"[Title/Abstract] OR "strokes cardiovascular"[Title/Abstract] OR "Myocardial Infarct"[Title/Abstract] OR "infarct myocardial"[Title/Abstract] OR "infarcts myocardial"[Title/Abstract] OR "Myocardial Infarcts"[Title/Abstract] OR "Heart Attack"[Title/Abstract] OR "Heart Attacks"[Title/Abstract] OR "acute coronary syndrome"[MeSH Terms] OR "angina pectoris"[MeSH Terms] OR "acute coronary syndrome"[Title/Abstract] OR "angina pectoris"[Title/Abstract] OR "Heart Arrest"[MeSH Terms] OR "Heart Arrest"[Title/Abstract] OR "arrest heart"[Title/Abstract] OR "Cardiac Arrest"[Title/Abstract] OR "arrest cardiac"[Title/Abstract] OR "Asystole"[Title/Abstract] OR "Asystoles"[Title/Abstract] OR "Cardiopulmonary Arrest"[Title/Abstract] OR "arrest cardiopulmonary"[Title/Abstract] OR "death, sudden, cardiac"[MeSH Terms] OR "death sudden cardiac"[Title/Abstract] OR "Sudden Cardiac Death"[Title/Abstract] OR "cardiac death sudden"[Title/Abstract] OR "death sudden cardiac"[Title/Abstract] OR "Cardiac Sudden Death"[Title/Abstract] OR "death cardiac sudden"[Title/Abstract] OR "sudden death cardiac"[Title/Abstract] OR "Sudden Cardiac Arrest"[Title/Abstract] OR "arrest sudden cardiac"[Title/Abstract] OR "cardiac arrest sudden"[Title/Abstract] OR "cardiovascular mortality"[Title/Abstract] OR "cardiovascular death"[Title/Abstract] OR "stroke"[MeSH Terms] OR

	"Cerebrovascular Accident"[Text Word] OR "Strokes"[Title/Abstract] OR "Cerebrovascular Accident"[Title/Abstract] OR "Cerebrovascular Accidents"[Title/Abstract] OR "cva cerebrovascular accident"[Title/Abstract] OR "Cerebrovascular Apoplexy"[Title/Abstract] OR "apoplexy cerebrovascular"[Title/Abstract] OR "vascular accident brain"[Title/Abstract] OR "Brain Vascular Accident"[Title/Abstract] OR "Brain Vascular Accidents"[Title/Abstract] OR "Cerebrovascular Stroke"[Title/Abstract] OR "Cerebrovascular Strokes"[Title/Abstract] OR "stroke cerebrovascular"[Title/Abstract] OR "strokes cerebrovascular"[Title/Abstract] OR "Apoplexy"[Title/Abstract] OR "Cerebral Stroke"[Title/Abstract] OR "Cerebral Strokes"[Title/Abstract] OR "stroke cerebral"[Title/Abstract] OR "strokes cerebral"[Title/Abstract] OR "stroke acute"[Title/Abstract] OR "Acute Stroke"[Title/Abstract] OR "Acute Strokes"[Title/Abstract] OR "strokes acute"[Title/Abstract] OR "cerebrovascular accident acute"[Title/Abstract] OR "Acute Cerebrovascular Accident"[Title/Abstract] OR "Acute Cerebrovascular Accidents"[Title/Abstract] OR "cerebrovascular accidents acute"[Title/Abstract] OR "brain attack"[Title/Abstract] OR "brain infarction"[MeSH Terms] OR "brain infarction"[Title/Abstract] OR "Brain Infarctions"[Title/Abstract] OR "infarction brain"[Title/Abstract] OR "infarctions brain"[Title/Abstract] OR "Brain Infarct"[Title/Abstract] OR "Brain Infarcts"[Title/Abstract] OR "infarct brain"[Title/Abstract] OR "infarcts brain"[Title/Abstract] OR "Anterior Circulation Brain Infarction"[Title/Abstract] OR "venous infarction brain"[Title/Abstract] OR "Brain Venous Infarction"[Title/Abstract] OR "venous infarctions brain"[Title/Abstract] OR "Venous Brain Infarctions"[Title/Abstract] OR "Anterior Cerebral Circulation Infarction"[Title/Abstract] OR "brain infarction posterior circulation"[Title/Abstract] OR "Posterior Circulation Brain Infarction"[Title/Abstract] OR "Ischemic Stroke"[MeSH Terms] OR "Ischemic Stroke"[Title/Abstract] OR "carotid artery diseases"[MeSH Terms] OR "carotid artery disease"[Title/Abstract] OR "cerebral small vessel diseases"[MeSH Terms] OR "cerebral small vessel disease"[Title/Abstract] OR "intracranial artery disease"[Title/Abstract] OR "cerebral arterial diseases"[MeSH Terms] OR "intracranial arteriosclerosis"[MeSH Terms] OR "arteriosclerosis intracranial"[Title/Abstract] OR "Intracranial Atherosclerosis"[Title/Abstract] OR "atherosclerosis intracranial"[Title/Abstract] OR "Cerebral Arteriosclerosis"[Title/Abstract] OR "arteriosclerosis cerebral"[Title/Abstract] OR "Cerebral Atherosclerosis"[Title/Abstract] OR "atherosclerosis cerebral"[Title/Abstract] OR "intracranial embolism and thrombosis"[MeSH Terms] OR "Cerebral Embolism and Thrombosis"[Title/Abstract] OR "cerebral infarction"[MeSH Terms] OR "cerebral ischemia"[Title/Abstract] OR "cerebral ischaemia"[Title/Abstract] OR "brain ischemia"[MeSH Terms] OR "brain ischaemia"[Title/Abstract]) AND ("diet, vegetarian"[MeSH Terms] OR "diet vegetarian"[Title/Abstract] OR "diets vegetarian"[Title/Abstract] OR "Vegetarian Diets"[Title/Abstract] OR "Vegetarian Diet"[Title/Abstract] OR "lacto vegetarian diet"[Title/Abstract] OR "lacto vegetarian diet"[Title/Abstract] OR "Lacto-Vegetarian Diets"[Title/Abstract] OR "plant based diet"[Title/Abstract] OR "diets plant based"[Title/Abstract] OR "plant based diet"[Title/Abstract] OR "Plant-Based Diets"[Title/Abstract] OR "diet plant based"[Title/Abstract] OR "diet plant based"[Title/Abstract] OR "plant based nutrition"[Title/Abstract] OR "nutrition plant based"[Title/Abstract] OR "plant based nutrition"[Title/Abstract] OR "lacto ovo vegetarian diet"[Title/Abstract] OR "diet lacto ovo vegetarian"[Title/Abstract] OR "diets lacto ovo vegetarian"[Title/Abstract] OR "lacto ovo vegetarian diet"[Title/Abstract] OR "Lacto-Ovo Vegetarian Diets"[Title/Abstract] OR "Vegetarianism"[Title/Abstract] OR "Vegetarians"[MeSH Terms] OR "Vegetarian"[Title/Abstract] OR "Vegetarians"[Title/Abstract] OR

	"Vegetarian"[Title/Abstract] OR "lacto ovo vegetarians"[Title/Abstract] OR "lacto ovo vegetarians"[Title/Abstract] OR "Lacto-Ovo Vegetarian"[Title/Abstract] OR "vegetarian lacto ovo"[Title/Abstract] OR "vegetarians lacto ovo"[Title/Abstract] OR "ovo lacto vegetarians"[Title/Abstract] OR "ovo lacto vegetarians"[Title/Abstract] OR "Ovo-Lacto Vegetarian"[Title/Abstract] OR "diet, vegan"[MeSH Terms] OR "diet vegan"[Title/Abstract] OR "diets vegan"[Title/Abstract] OR "Vegan Diets"[Title/Abstract] OR "Vegan Diet"[Title/Abstract] OR "Veganism"[Title/Abstract] OR "vegans"[MeSH Terms] OR "vegans"[Title/Abstract] OR "vegan"[Title/Abstract] OR "seventh day adventists"[Title/Abstract] OR "seventh day adventists"[Title/Abstract] OR "Seventh-Day Adventist"[Title/Abstract] OR "diet, western"[MeSH Terms] OR "diet western"[Title/Abstract] OR "diet, mediterranean"[MeSH Terms] OR "mediterranean diet"[Title/Abstract] OR "mediterranean diet"[Title/Abstract] OR "Feeding Behavior"[MeSH Terms] OR "Feeding Behavior"[Title/Abstract] OR "behavior feeding"[Title/Abstract] OR "Feeding Behaviors"[Title/Abstract] OR "Eating Behavior"[Title/Abstract] OR "behavior eating"[Title/Abstract] OR "Eating Behaviors"[Title/Abstract] OR "feeding related behavior"[Title/Abstract] OR "feeding related behavior"[Title/Abstract] OR "Feeding-Related Behaviors"[Title/Abstract] OR "Feeding Patterns"[Title/Abstract] OR "Feeding Pattern"[Title/Abstract] OR "pattern feeding"[Title/Abstract] OR "Food Habits"[Title/Abstract] OR "Food Habit"[Title/Abstract] OR "habit food"[Title/Abstract] OR "Eating Habits"[Title/Abstract] OR "Eating Habit"[Title/Abstract] OR "habit eating"[Title/Abstract] OR "Dietary Habits"[Title/Abstract] OR "Dietary Habit"[Title/Abstract] OR "habit dietary"[Title/Abstract] OR "Diet Habits"[Title/Abstract] OR "Diet Habit"[Title/Abstract] OR "habit diet"[Title/Abstract] OR "habits diet"[Title/Abstract] OR "dietary pattern"[Title/Abstract] OR "eating pattern"[Title/Abstract] OR "food pattern"[Title/Abstract])<br>AND "systematic"[filter] OR "meta-analysis"[pt] OR "meta-analysis as topic"[mh] OR "meta analy*"[tw] OR metanaly*[tw] OR metaanaly*[tw] OR "met analy*"[tw] OR "integrative research"[tiab] OR "integrative review*"[tiab] OR "integrative overview*"[tiab] OR "research integration*"[tiab] OR "research overview*"[tiab] OR "collaborative review*"[tiab] OR "collaborative overview*"[tiab] OR "systematic review"[pt] OR "systematic reviews as topic"[mh] OR "systematic review*"[tiab] OR "technology assessment*"[tiab] OR "technology overview*"[tiab] OR "technology appraisal*"[tiab] OR "Technology Assessment, Biomedical"[mh] OR HTA[tiab] OR HTAs[tiab] OR "comparative efficacy"[tiab] OR "comparative effectiveness"[tiab] OR "outcomes research"[tiab] OR "indirect comparison*"[tiab] OR "Bayesian comparison"[tiab] OR (("indirect treatment"[tiab] OR "mixed-treatment"[tiab]) AND comparison*[tiab]) OR Embase*[tiab] OR Cinahl*[tiab] OR "systematic overview*"[tiab] OR "methodological overview*"[tiab] OR "methodologic overview*"[tiab] OR "methodological review*"[tiab] OR "methodologic review*"[tiab] OR "quantitative review*"[tiab] OR "quantitative overview*"[tiab] OR "quantitative synthes*"[tiab] OR "pooled analy*"[tiab] OR Cochrane[tiab] OR Medline[tiab] OR Pubmed[tiab] OR Medlars[tiab] OR handsearch*[tiab] OR "hand search*"[tiab] OR "meta-regression*"[tiab] OR metaregression*[tiab] OR "data synthes*"[tiab] OR "data extraction"[tiab] OR "data abstraction*"[tiab] OR "mantel haenszel"[tiab] OR peto[tiab] OR "der-simonian"[tiab] OR dersimonian[tiab] OR "fixed effect*"[tiab] OR "multiple treatment comparison"[tiab] OR "mixed treatment meta-analys*"[tiab] OR "umbrella review*"[tiab] OR (("multiple paramet*"[tiab]) AND ("evidence synthesis"[tiab])) OR (("multi-paramet*"[tiab]) AND ("evidence synthesis"[tiab])) OR ((multiparameter*[tiab]) AND ("evidence synthesis"[tiab])) OR "Cochrane Database

	Syst Rev"[Journal] OR "health technology assessment winchester, england"[Journal] OR "Evid Rep Technol Assess (Full Rep)"[Journal] OR "Evid Rep Technol Assess (Summ)"[Journal] OR "Int J Technol Assess Health Care"[Journal] OR "GMS Health Technol Assess"[Journal] OR "Health Technol Assess (Rockv)"[Journal] OR "Health Technol Assess Rep"[Journal]
Scopus	TITLE-ABS (("myocardial ischemia" OR "Ischemia, Myocardial" OR "Ischemias, Myocardial" OR "Myocardial Ischemias" OR "Ischemic Heart Disease" OR "Heart Disease, Ischemic" OR "Disease, Ischemic Heart" OR "Diseases, Ischemic Heart" OR "Heart Diseases, Ischemic" OR "Ischemic Heart Diseases" OR "coronary disease" OR "Coronary Diseases" OR "Disease, Coronary" OR "Diseases, Coronary" OR "Coronary Heart Disease" OR "Coronary Heart Diseases" OR "Disease, Coronary Heart" OR "Diseases, Coronary Heart" OR "Heart Disease, Coronary" OR "Heart Diseases, Coronary" OR "Coronary Heart Disease" OR "Myocardial Infarction" OR "Infarction, Myocardial" OR "Infarctions, Myocardial" OR "Myocardial Infarctions" OR "Cardiovascular Stroke" OR "Cardiovascular Strokes" OR "Stroke, Cardiovascular" OR "Strokes, Cardiovascular" OR "Myocardial Infarct" OR "Infarct, Myocardial" OR "Infarcts, Myocardial" OR "Myocardial Infarcts" OR "Heart Attack" OR "Heart Attacks" OR "acute coronary syndrome" OR "angina pectoris" OR "acute coronary syndrome" OR "angina pectoris" OR "Heart Arrest" OR "Arrest, Heart" OR "Cardiac Arrest" OR "Arrest, Cardiac" OR "Asystole" OR "Asystoles" OR "Cardiopulmonary Arrest" OR "Arrest, Cardiopulmonary" OR "Death, Sudden, Cardiac" OR "Sudden Cardiac Death" OR "Cardiac Death, Sudden" OR "Death, Sudden Cardiac" OR "Cardiac Sudden Death" OR "Death, Cardiac Sudden" OR "Sudden Death, Cardiac" OR "Sudden Cardiac Arrest" OR "Arrest, Sudden Cardiac" OR "Cardiac Arrests, Sudden" OR "Cardiac Arrest, Sudden" OR "cardiovascular mortality" OR "cardiovascular death" OR "stroke" OR "cerebrovascular accident" OR "Strokes" OR "Cerebrovascular Accident" OR "Cerebrovascular Accidents" OR "CVA (Cerebrovascular Accident)" OR "CVAs (Cerebrovascular Accident)" OR "Cerebrovascular Apoplexy" OR "Apoplexy, Cerebrovascular" OR "Vascular Accident, Brain" OR "Brain Vascular Accident" OR "Brain Vascular Accidents" OR "Vascular Accidents, Brain" OR "Cerebrovascular Stroke" OR "Cerebrovascular Strokes" OR "Stroke, Cerebrovascular" OR "Strokes, Cerebrovascular" OR "Apoplexy" OR "Cerebral Stroke" OR "Cerebral Strokes" OR "Stroke, Cerebral" OR "Strokes, Cerebral" OR "Stroke, Acute" OR "Acute Stroke" OR "Acute Strokes" OR "Strokes, Acute" OR "Cerebrovascular Accident, Acute" OR "Acute Cerebrovascular Accident" OR "Acute Cerebrovascular Accidents" OR "Cerebrovascular Accidents, Acute" OR "brain attack" OR "brain infarction" OR "Brain Infarctions" OR "Infarction, Brain" OR "Infarctions, Brain" OR "Brain Infarct" OR "Brain Infarcts" OR "Infarct, Brain" OR "Infarcts, Brain" OR "Anterior Circulation Brain Infarction" OR "Infarction, Brain, Anterior Circulation" OR "Infarction, Anterior Circulation, Brain" OR "Anterior Circulation Infarction, Brain" OR "Brain Infarction, Anterior Circulation" OR "Venous Infarction, Brain" OR "Brain Venous Infarction" OR "Brain Venous Infarctions" OR "Infarction, Brain Venous" OR "Infarctions, Brain Venous" OR "Venous Infarctions, Brain" OR "Brain Infarction, Venous" OR "Brain Infarctions, Venous" OR "Infarction, Venous Brain" OR "Infarctions, Venous Brain" OR "Venous Brain Infarction" OR "Venous Brain Infarctions" OR "Anterior Cerebral Circulation Infarction" OR "Infarction, Anterior Cerebral Circulation" OR "Brain Infarction, Posterior Circulation" OR "Posterior Circulation Infarction, Brain" OR "Posterior Circulation Brain Infarction" OR "Infarction, Brain, Posterior Circulation" OR "Infarction, Posterior Circulation, Brain" OR "Hemorrhagic Stroke" OR "Ischemic Stroke" OR "carotid artery diseases" OR "cerebral small vessel diseases"

	OR "intracranial artery disease" OR "cerebral arterial diseases" OR "cerebral artery disease" OR "intracranial arteriosclerosis" OR "intracranial embolism and thrombosis" OR "intracranial hemorrhages" OR "intracranial hemorrhage" OR "intracranial haemorrhage" OR "cerebral haemorrhage" OR "cerebral haemorrhage" OR "intracerebral hemorrhage" OR "intracerebral haemorrhage" OR "subarachnoid haemorrhage" OR "subarachnoid haemorrhage" OR "cerebral infarction" OR "cerebral ischemia" OR "cerebral ischaemia" OR "brain ischemia" OR "brain ischaemia" OR "brain hemorrhage" OR "brain haemorrhage") AND ("diet, vegetarian" OR "Diets, Vegetarian" OR "Vegetarian Diets" OR "Vegetarian Diet" OR "Lacto-Vegetarian Diet" OR "Diet, Lacto-Vegetarian" OR "Diets, Lacto-Vegetarian" OR "Lacto Vegetarian Diet" OR "Lacto-Vegetarian Diets" OR "Plant-Based Diet" OR "Diets, Plant-Based" OR "Plant Based Diet" OR "Plant-Based Diets" OR "Diet, Plant-Based" OR "Diet, Plant Based" OR "Plant-Based Nutrition" OR "Nutrition, Plant-Based" OR "Plant Based Nutrition" OR "Lacto-Ovo Vegetarian Diet" OR "Diet, Lacto-Ovo Vegetarian" OR "Diets, Lacto-Ovo Vegetarian" OR "Lacto Ovo Vegetarian Diet" OR "Lacto-Ovo Vegetarian Diets" OR "Vegetarian Diet, Lacto-Ovo" OR "Vegetarian Diets, Lacto-Ovo" OR "Vegetarianism" OR "Vegetarians" OR vegetarian OR "Lacto-Ovo Vegetarians" OR "Lacto Ovo Vegetarians" OR "Lacto-Ovo Vegetarian" OR "Vegetarian, Lacto-Ovo" OR "Vegetarians, Lacto-Ovo" OR "Ovo-Lacto Vegetarians" OR "Ovo Lacto Vegetarians" OR "Ovo-Lacto Vegetarian" OR "Vegetarian, Ovo-Lacto" OR "Vegetarians, Ovo-Lacto" OR "Diet, Vegan" OR "Diets, Vegan" OR "Vegan Diets" OR "Vegan Diet" OR "Veganism" OR "vegans" OR vegan OR "Seventh-Day Adventists" OR "Adventist, Seventh-Day" OR "Adventists, Seventh-Day" OR "Seventh Day Adventists" OR "Seventh-Day Adventist" OR "mediterranean diet" OR "mediterranean diets" OR "Diet, Western" OR "Diet, Western" OR "Feeding Behavior" OR "Feeding Behavior" OR "Behavior, Feeding" OR "Feeding Behaviors" OR "Eating Behavior" OR "Behavior, Eating" OR "Eating Behaviors" OR "Feeding-Related Behavior" OR "Behavior, Feeding-Related" OR "Feeding Related Behavior" OR "Feeding-Related Behaviors" OR "Feeding Patterns" OR "Feeding Pattern" OR "Pattern, Feeding" OR "Food Habits" OR "Food Habit" OR "Habit, Food" OR "Eating Habits" OR "Eating Habit" OR "Habit, Eating" OR "Dietary Habits" OR "Dietary Habit" OR "Habit, Dietary" OR "Diet Habits" OR "Diet Habit" OR "Habit, Diet" OR "Habits, Diet" OR "dietary pattern" OR "eating pattern" OR "food pattern"))
	AND
	TITLE-ABS-KEY((systematic* W/3 (review* OR overview*)) OR (methodologic* W/3 (review* OR overview*))) OR TITLE-ABS-KEY((quantitative W/3 (review* OR overview* OR synthes*)) OR (research W/3 (integrati* OR overview*))) OR TITLE-ABS-KEY((integrative W/3 (review* OR overview*)) OR (collaborative W/3 (review* OR overview*)) OR (pool* W/3 analy*)) OR TITLE-ABS-KEY("data synthes*" OR "data extraction*" OR "data abstraction*") OR TITLE-ABS-KEY(handsearch* OR "hand search*") OR TITLE-ABS-KEY("mantel haenszel" OR peto OR "der simonian" OR dersimonian OR "fixed effect*" OR "latin square*") OR TITLE-ABS-KEY("met analy*" OR metanaly* OR "technology assessment*" OR HTA OR HTAs OR "technology overview*" OR "technology appraisal*") OR TITLE-ABS-KEY("meta regression*" OR metaregression*) OR TITLE-ABS-KEY(meta-analy* OR metaanaly* OR "systematic review*" OR "biomedical technology assessment*" OR "bio-medical technology assessment*") OR TITLE-ABS-KEY(medline OR cochrane OR pubmed OR medlars OR embase OR cinahl) OR SRCTITLE(cochrane OR (health W/2 "technology assessment") OR "evidence report") OR TITLE-ABS-KEY(comparative W/3 (efficacy

	OR effectiveness)) OR TITLE-ABS-KEY("outcomes research" OR "relative effectiveness") OR TITLE-ABS-KEY((indirect OR "indirect treatment" OR mixed-treatment OR bayesian) W/3 comparison*) OR TITLE-ABS-KEY(multi* W/3 treatment W/3 comparison*) OR TITLE-ABS-KEY(mixed W/3 treatment W/3 (meta-analy* OR metaanaly*)) OR TITLE-ABS-KEY("umbrella review*") OR TITLE-ABS-KEY(multi* W/2 paramet* W/2 evidence W/2 synthesis) OR TITLE-ABS-KEY(multiparamet* W/2 evidence W/2 synthesis) OR TITLE-ABS-KEY(multi-paramet* W/2 evidence W/2 synthesis)
Embase	"myocardial ischemia".tw. or "heart muscle ischemia"/ or ("Myocardial Ischemias" or "Ischemic Heart Disease" or "Ischemic Heart Diseases").tw. or "coronary artery disease"/ or "coronary disease".tw. or ("Coronary Diseases" or "Coronary Heart Diseases" or "Coronary Heart Disease").tw. or ("Coronary Artery Diseases" or "Left Main Coronary Artery Disease" or "Left Main Disease" or "Left Main Diseases" or "Left Main Coronary Disease" or "Coronary Arteriosclerosis" or "Coronary Arterioscleroses" or "Coronary Atheroscleroses" or "Coronary Atherosclerosis").tw. or "Coronary Heart Disease".tw. or "ischemic heart disease"/ or "myocardial infarction".tw. or "heart infarction"/ or ("Myocardial Infarctions" or "Cardiovascular Stroke" or "Cardiovascular Strokes" or "Myocardial Infarct" or "Myocardial Infarcts" or "Heart Attack" or "Heart Attacks").tw. or "angina pectoris".tw. or "angina pectoris"/ or "acute coronary syndrome".tw. or "heart arrest".tw. or "heart arrest"/ or ("Cardiac Arrest" or Asystole or Asystoles or "Cardiopulmonary Arrest").tw. or "death sudden cardiac".tw. or "sudden cardiac death"/ or ("Sudden Cardiac Death" or "Cardiac Sudden Death" or "Sudden Cardiac Arrest").tw. or "cardiovascular mortality".tw. or "cardiovascular mortality"/ or stroke.tw. or "cerebrovascular accident"/ or (Strokes or "Cerebrovascular Accident" or "Cerebrovascular Accidents" or "Cerebrovascular Apoplexy" or "Brain Vascular Accident" or "Brain Vascular Accidents" or "Cerebrovascular Stroke" or "Cerebrovascular Strokes" or Apoplexy or "Cerebral Stroke" or "Cerebral Strokes" or "Acute Stroke" or "Acute Strokes" or "Acute Cerebrovascular Accident" or "Acute Cerebrovascular Accidents" or "brain attack").tw. or "brain infarction".tw. or "brain infarction"/ or ("Brain Infarctions" or "Brain Infarct" or "Brain Infarcts" or "Anterior Circulation Brain Infarction" or "Brain Venous Infarction" or "Brain Venous Infarctions" or "Venous Brain Infarction" or "Venous Brain Infarctions" or "Anterior Cerebral Circulation Infarction" or "Posterior Circulation Brain Infarction").tw. or "ischemic stroke".tw. or "ischemic stroke"/ or "brain ischemia"/ or "carotid artery disease".tw. or "carotid artery disease"/ or "cerebral small vessel disease".tw. or "intracranial artery disease".tw. or "cerebral arterial diseases".tw. or "cerebral artery disease"/ or "intracranial arteriosclerosis".tw. or "brain atherosclerosis"/ or ("Intracranial Arterioscleroses" or "Intracranial Atherosclerosis" or "Intracranial Atheroscleroses" or "Cerebral Arteriosclerosis" or "Cerebral Arterioscleroses" or "Cerebral Atherosclerosis" or "Cerebral Atheroscleroses").tw. or "intracranial embolism".tw. or "brain embolism"/ or ("Cerebral Embolism and Thrombosis" or "Brain Embolism and Thrombosis").tw. or ("cerebral infarction" or "cerebral ischaemia" or "brain ischaemia").tw.
	AND
	"vegetarian diet".tw. or "vegetarian diet"/ or ("Vegetarian Diets" or "Vegetarian Diet" or "Lacto-Vegetarian Diet" or "Lacto Vegetarian Diet" or "Lacto-Vegetarian Diets" or "Plant-Based Diet" or "Plant Based Diet" or "Plant-Based Diets" or "Plant-Based Nutrition" or "Plant Based Nutrition" or "plant based diet" or "Lacto-Ovo Vegetarian Diet" or "Lacto Ovo Vegetarian Diet" or "Lacto-Ovo Vegetarian Diets" or Vegetarianism).tw. or vegetarians.tw. or vegetarian/ or vegetarian.tw. or ("Lacto-Ovo Vegetarians" or "Lacto Ovo Vegetarians" or "Lacto-Ovo Vegetarian" or "Ovo-Lacto

	Vegetarians" or "Ovo Lacto Vegetarians" or "Ovo-Lacto Vegetarian").tw. or "vegan diet"/ or "Diet, Vegan".tw. or "Vegan Diets".tw. or "Vegan Diet".tw. or Veganism.tw. or vegans/ or vegans.tw. or vegan.tw. or "Seventh Day Adventists".tw. or "Seventh-day Adventist"/ or "Seventh-Day Adventists".tw. or "Seventh-Day Adventist".tw. or "Western diet"/ or "Diet, Western".tw. or "Mediterranean diet"/ or "Mediterranean diet".tw. or "feeding behavior".tw. or "feeding behavior"/ or "Feeding Behaviors".tw. or "Eating Behavior".tw. or "Eating Behaviors".tw. or "Feeding-Related Behavior".tw. or "Feeding Related Behavior".tw. or "Feeding-Related Behaviors".tw. or "Feeding Patterns".tw. or "Feeding Pattern".tw. or "Food Habits".tw. or "Food Habit".tw. or "Eating Habits".tw. or "Eating Habit".tw. or "Dietary Habits".tw. or "Dietary Habit".tw. or "Diet Habits".tw. or "Diet Habit".tw. or "eating habit"/ or "eating habit".tw. or "dietary pattern".tw. or "dietary pattern".tw. or "eating pattern".tw. or "food pattern".tw.
	AND
	1. (systematic review or meta-analysis).pt.<br>2. meta-analysis/ or systematic review/ or systematic reviews as topic/ or meta-analysis as topic/ or "meta analysis (topic)"/ or "systematic review (topic)"/ or exp technology assessment, biomedical/ or network meta-analysis/<br>3. ((systematic* adj3 (review* or overview*)) or (methodologic* adj3 (review* or overview*))).ti,ab,kf.<br>4. ((quantitative adj3 (review* or overview* or synthes*)) or (research adj3 (integrati* or overview*))).ti,ab,kf.<br>5. ((integrative adj3 (review* or overview*)) or (collaborative adj3 (review* or overview*)) or (pool* adj3 analy*)).ti,ab,kf.<br>6. (data synthes* or data extraction* or data abstraction*).ti,ab,kf.<br>7. (handsearch* or hand search*).ti,ab,kf.<br>8. (mantel haenszel or peto or der simonian or dersimonian or fixed effect* or latin square*).ti,ab,kf.<br>9. (met analy* or metanaly* or technology assessment* or HTA or HTAs or technology overview* or technology appraisal*).ti,ab,kf.<br>10. (meta regression* or metaregression*).ti,ab,kf.<br>11. (meta-analy* or metaanaly* or systematic review* or biomedical technology assessment* or bio-medical technology assessment*).mp,hw.<br>12. (medline or cochrane or pubmed or medlars or embase or cinahl).ti,ab,hw.<br>13. (cochrane or (health adj2 technology assessment) or evidence report).jw.<br>14. (comparative adj3 (efficacy or effectiveness)).ti,ab,kf.<br>15. (outcomes research or relative effectiveness).ti,ab,kf.<br>16. ((indirect or indirect treatment or mixed-treatment or bayesian) adj3 comparison*).ti,ab,kf.<br>17. (meta-analysis or systematic review).md.<br>18. (multi* adj3 treatment adj3 comparison*).ti,ab,kf.<br>19. (mixed adj3 treatment adj3 (meta-analy* or metaanaly*)).ti,ab,kf.<br>20. umbrella review*.ti,ab,kf.<br>21. (multi* adj2 paramet* adj2 evidence adj2 synthesis).ti,ab,kf.<br>22. (multiparamet* adj2 evidence adj2 synthesis).ti,ab,kf.<br>23. (multi-paramet* adj2 evidence adj2 synthesis).ti,ab,kf.<br>24. or/1-23
CINHAL	((MH "myocardial ischemia+") OR (TI "myocardial ischaemia" OR AB "myocardial ischaemia") OR (TI "ischemia myocardial" OR AB "ischemia myocardial") OR (TI "ischemias myocardial" OR AB "ischemias myocardial") OR (TI "Myocardial Ischemias" OR AB "Myocardial Ischemias") OR (TI "Ischemic Heart Disease" OR AB

	"Ischemic Heart Disease") OR (TI "heart disease ischemic" OR AB "heart disease ischemic") OR (TI "disease ischemic heart" OR AB "disease ischemic heart") OR (TI "diseases ischemic heart" OR AB "diseases ischemic heart") OR (TI "heart diseases ischemic" OR AB "heart diseases ischemic") OR (TI "Ischemic Heart Diseases" OR AB "Ischemic Heart Diseases") OR (MH "coronary disease+") OR (TI "coronary disease" OR AB "coronary disease") OR (TI "Coronary Diseases" OR AB "Coronary Diseases") OR (TI "disease coronary" OR AB "disease coronary") OR (TI "diseases coronary" OR AB "diseases coronary") OR (TI "Coronary Heart Disease" OR AB "Coronary Heart Disease") OR (TI "Coronary Heart Diseases" OR AB "Coronary Heart Diseases") OR (TI "disease coronary heart" OR AB "disease coronary heart") OR (TI "diseases coronary heart" OR AB "diseases coronary heart") OR (TI "heart disease coronary" OR AB "heart disease coronary") OR (TI "heart diseases coronary" OR AB "heart diseases coronary") OR (MH "coronary artery disease+") OR (TI "coronary artery disease" OR AB "coronary artery disease") OR (TI "artery disease coronary" OR AB "artery disease coronary") OR (TI "artery diseases coronary" OR AB "artery diseases coronary") OR (TI "Coronary Artery Diseases" OR AB "Coronary Artery Diseases") OR (TI "Left Main Coronary Artery Disease" OR AB "Left Main Coronary Artery Disease") OR (TI "Left Main Disease" OR AB "Left Main Disease") OR (TI "Left Main Diseases" OR AB "Left Main Diseases") OR (TI "Left Main Coronary Disease" OR AB "Left Main Coronary Disease") OR (TI "Coronary Arteriosclerosis" OR AB "Coronary Arteriosclerosis") OR (TI "atherosclerosis coronary" OR AB "atherosclerosis coronary") OR (TI "Coronary Atherosclerosis" OR AB "Coronary Atherosclerosis") OR (TI "arteriosclerosis coronary" OR AB "arteriosclerosis coronary") OR (TI "Coronary Heart Disease" OR AB "Coronary Heart Disease") OR (MH "Myocardial Infarction+") OR (TI "Myocardial Infarction" OR AB "Myocardial Infarction") OR (TI "infarction myocardial" OR AB "infarction myocardial") OR (TI "infarctions myocardial" OR AB "infarctions myocardial") OR (TI "Myocardial Infarctions" OR AB "Myocardial Infarctions") OR (TI "Cardiovascular Stroke" OR AB "Cardiovascular Stroke") OR (TI "stroke cardiovascular" OR AB "stroke cardiovascular") OR (TI "strokes cardiovascular" OR AB "strokes cardiovascular") OR (TI "Myocardial Infarct" OR AB "Myocardial Infarct") OR (TI "infarct myocardial" OR AB "infarct myocardial") OR (TI "infarcts myocardial" OR AB "infarcts myocardial") OR (TI "Myocardial Infarcts" OR AB "Myocardial Infarcts") OR (TI "Heart Attack" OR AB "Heart Attack") OR (TI "Heart Attacks" OR AB "Heart Attacks") OR (MH "acute coronary syndrome+") OR (MH "angina pectoris+") OR (TI "acute coronary syndrome" OR AB "acute coronary syndrome") OR (TI "angina pectoris" OR AB "angina pectoris") OR (MH "Heart Arrest+") OR (TI "Heart Arrest" OR AB "Heart Arrest") OR (TI "arrest heart" OR AB "arrest heart") OR (TI "Cardiac Arrest" OR AB "Cardiac Arrest") OR (TI "arrest cardiac" OR AB "arrest cardiac") OR (TI Asystole OR AB Asystole) OR (TI Asystoles OR AB Asystoles) OR (TI "Cardiopulmonary Arrest" OR AB "Cardiopulmonary Arrest") OR (TI "arrest cardiopulmonary" OR AB "arrest cardiopulmonary") OR (MH "death, sudden, cardiac+") OR (TI "death sudden cardiac" OR AB "death sudden cardiac") OR (TI "Sudden Cardiac Death" OR AB "Sudden Cardiac Death") OR (TI "cardiac death sudden" OR AB "cardiac death sudden") OR (TI "death sudden cardiac" OR AB "death sudden cardiac") OR (TI "Cardiac Sudden Death" OR AB "Cardiac Sudden Death") OR (TI "death cardiac sudden" OR AB "death cardiac sudden") OR (TI "sudden death cardiac" OR AB "sudden death cardiac") OR (TI "Sudden Cardiac Arrest" OR AB "Sudden Cardiac Arrest") OR (TI "arrest sudden cardiac" OR AB "arrest sudden cardiac") OR (TI "cardiac arrest sudden" OR AB "cardiac arrest sudden") OR (TI "cardiovascular mortality" OR AB "cardiovascular mortality") OR (TI "cardiovascular death" OR AB "cardiovascular death") OR (MH stroke+) OR "Cerebrovascular Accident" OR (TI Strokes OR AB Strokes) OR (TI

	"Cerebrovascular Accident" OR AB "Cerebrovascular Accident") OR (TI "Cerebrovascular Accidents" OR AB "Cerebrovascular Accidents") OR (TI "cva cerebrovascular accident" OR AB "cva cerebrovascular accident") OR (TI "Cerebrovascular Apoplexy" OR AB "Cerebrovascular Apoplexy") OR (TI "apoplexy cerebrovascular" OR AB "apoplexy cerebrovascular") OR (TI "vascular accident brain" OR AB "vascular accident brain") OR (TI "Brain Vascular Accident" OR AB "Brain Vascular Accident") OR (TI "Brain Vascular Accidents" OR AB "Brain Vascular Accidents") OR (TI "Cerebrovascular Stroke" OR AB "Cerebrovascular Stroke") OR (TI "Cerebrovascular Strokes" OR AB "Cerebrovascular Strokes") OR (TI "stroke cerebrovascular" OR AB "stroke cerebrovascular") OR (TI "strokes cerebrovascular" OR AB "strokes cerebrovascular") OR (TI Apoplexy OR AB Apoplexy) OR (TI "Cerebral Stroke" OR AB "Cerebral Stroke") OR (TI "Cerebral Strokes" OR AB "Cerebral Strokes") OR (TI "stroke cerebral" OR AB "stroke cerebral") OR (TI "strokes cerebral" OR AB "strokes cerebral") OR (TI "stroke acute" OR AB "stroke acute") OR (TI "Acute Stroke" OR AB "Acute Stroke") OR (TI "Acute Strokes" OR AB "Acute Strokes") OR (TI "strokes acute" OR AB "strokes acute") OR (TI "cerebrovascular accident acute" OR AB "cerebrovascular accident acute") OR (TI "Acute Cerebrovascular Accident" OR AB "Acute Cerebrovascular Accident") OR (TI "Acute Cerebrovascular Accidents" OR AB "Acute Cerebrovascular Accidents") OR (TI "cerebrovascular accidents acute" OR AB "cerebrovascular accidents acute") OR (TI "brain attack" OR AB "brain attack") OR (MH "brain infarction+") OR (TI "brain infarction" OR AB "brain infarction") OR (TI "Brain Infarctions" OR AB "Brain Infarctions") OR (TI "infarction brain" OR AB "infarction brain") OR (TI "infarctions brain" OR AB "infarctions brain") OR (TI "Brain Infarct" OR AB "Brain Infarct") OR (TI "Brain Infarcts" OR AB "Brain Infarcts") OR (TI "infarct brain" OR AB "infarct brain") OR (TI "infarcts brain" OR AB "infarcts brain") OR (TI "Anterior Circulation Brain Infarction" OR AB "Anterior Circulation Brain Infarction") OR (TI "venous infarction brain" OR AB "venous infarction brain") OR (TI "Brain Venous Infarction" OR AB "Brain Venous Infarction") OR (TI "venous infarctions brain" OR AB "venous infarctions brain") OR (TI "Venous Brain Infarctions" OR AB "Venous Brain Infarctions") OR (TI "Anterior Cerebral Circulation Infarction" OR AB "Anterior Cerebral Circulation Infarction") OR (TI "brain infarction posterior circulation" OR AB "brain infarction posterior circulation") OR (TI "Posterior Circulation Brain Infarction" OR AB "Posterior Circulation Brain Infarction") OR (MH "Ischemic Stroke+") OR (TI "Ischemic Stroke" OR AB "Ischemic Stroke") OR (MH "carotid artery diseases+") OR (TI "carotid artery disease" OR AB "carotid artery disease") OR (MH "cerebral small vessel diseases+") OR (TI "cerebral small vessel disease" OR AB "cerebral small vessel disease") OR (TI "intracranial artery disease" OR AB "intracranial artery disease") OR (MH "cerebral arterial diseases+") OR (MH "intracranial arteriosclerosis+") OR (TI "arteriosclerosis intracranial" OR AB "arteriosclerosis intracranial") OR (TI "Intracranial Atherosclerosis" OR AB "Intracranial Atherosclerosis") OR (TI "atherosclerosis intracranial" OR AB "atherosclerosis intracranial") OR (TI "Cerebral Arteriosclerosis" OR AB "Cerebral Arteriosclerosis") OR (TI "arteriosclerosis cerebral" OR AB "arteriosclerosis cerebral") OR (TI "Cerebral Atherosclerosis" OR AB "Cerebral Atherosclerosis") OR (TI "atherosclerosis cerebral" OR AB "atherosclerosis cerebral") OR (MH "intracranial embolism and thrombosis+") OR (TI "Cerebral Embolism and Thrombosis" OR AB "Cerebral Embolism and Thrombosis") OR (MH "cerebral infarction+") OR (TI "cerebral ischemia" OR AB "cerebral ischemia") OR (TI "cerebral ischaemia" OR AB "cerebral ischaemia") OR (MH "brain ischemia+") OR (TI "brain ischaemia" OR AB "brain ischaemia")) AND ((MH "diet, vegetarian+") OR (TI "diet vegetarian" OR AB "diet vegetarian") OR (TI "diets vegetarian" OR AB "diets vegetarian") OR (TI

	"Vegetarian Diets" OR AB "Vegetarian Diets") OR (TI "Vegetarian Diet" OR AB "Vegetarian Diet") OR (TI "lacto vegetarian diet" OR AB "lacto vegetarian diet") OR (TI "lacto vegetarian diet" OR AB "lacto vegetarian diet") OR (TI "Lacto-Vegetarian Diets" OR AB "Lacto-Vegetarian Diets") OR (TI "plant based diet" OR AB "plant based diet") OR (TI "diets plant based" OR AB "diets plant based") OR (TI "plant based diet" OR AB "plant based diet") OR (TI "Plant-Based Diets" OR AB "Plant-Based Diets") OR (TI "diet plant based" OR AB "diet plant based") OR (TI "diet plant based" OR AB "diet plant based") OR (TI "plant based nutrition" OR AB "plant based nutrition") OR (TI "nutrition plant based" OR AB "nutrition plant based") OR (TI "plant based nutrition" OR AB "plant based nutrition") OR (TI "lacto ovo vegetarian diet" OR AB "lacto ovo vegetarian diet") OR (TI "diet lacto ovo vegetarian" OR AB "diet lacto ovo vegetarian") OR (TI "diets lacto ovo vegetarian" OR AB "diets lacto ovo vegetarian") OR (TI "lacto ovo vegetarian diet" OR AB "lacto ovo vegetarian diet") OR (TI "Lacto-Ovo Vegetarian Diets" OR AB "Lacto-Ovo Vegetarian Diets") OR (TI Vegetarianism OR AB Vegetarianism) OR (MH Vegetarians+) OR (TI Vegetarian OR AB Vegetarian) OR (TI Vegetarians OR AB Vegetarians) OR (TI Vegetarian OR AB Vegetarian) OR (TI "lacto ovo vegetarians" OR AB "lacto ovo vegetarians") OR (TI "lacto ovo vegetarians" OR AB "lacto ovo vegetarians") OR (TI "Lacto-Ovo Vegetarian" OR AB "Lacto-Ovo Vegetarian") OR (TI "vegetarian lacto ovo" OR AB "vegetarian lacto ovo") OR (TI "vegetarians lacto ovo" OR AB "vegetarians lacto ovo") OR (TI "ovo lacto vegetarians" OR AB "ovo lacto vegetarians") OR (TI "ovo lacto vegetarians" OR AB "ovo lacto vegetarians") OR (TI "Ovo-Lacto Vegetarian" OR AB "Ovo-Lacto Vegetarian") OR (MH "diet, vegan+") OR (TI "diet vegan" OR AB "diet vegan") OR (TI "diets vegan" OR AB "diets vegan") OR (TI "Vegan Diets" OR AB "Vegan Diets") OR (TI "Vegan Diet" OR AB "Vegan Diet") OR (TI Veganism OR AB Veganism) OR (MH vegans+) OR (TI vegans OR AB vegans) OR (TI vegan OR AB vegan) OR (TI "seventh day adventists" OR AB "seventh day adventists") OR (TI "seventh day adventists" OR AB "seventh day adventists") OR (TI "Seventh-Day Adventist" OR AB "Seventh-Day Adventist") OR (MH "diet, western+") OR (TI "diet western" OR AB "diet western") OR (MH "diet, mediterranean+") OR (TI "mediterranean diet" OR AB "mediterranean diet") OR (TI "mediterranean diet" OR AB "mediterranean diet") OR (MH "Feeding Behavior+") OR (TI "Feeding Behavior" OR AB "Feeding Behavior") OR (TI "behavior feeding" OR AB "behavior feeding") OR (TI "Feeding Behaviors" OR AB "Feeding Behaviors") OR (TI "Eating Behavior" OR AB "Eating Behavior") OR (TI "behavior eating" OR AB "behavior eating") OR (TI "Eating Behaviors" OR AB "Eating Behaviors") OR (TI "feeding related behavior" OR AB "feeding related behavior") OR (TI "feeding related behavior" OR AB "feeding related behavior") OR (TI "Feeding-Related Behaviors" OR AB "Feeding-Related Behaviors") OR (TI "Feeding Patterns" OR AB "Feeding Patterns") OR (TI "Feeding Pattern" OR AB "Feeding Pattern") OR (TI "pattern feeding" OR AB "pattern feeding") OR (TI "Food Habits" OR AB "Food Habits") OR (TI "Food Habit" OR AB "Food Habit") OR (TI "habit food" OR AB "habit food") OR (TI "Eating Habits" OR AB "Eating Habits") OR (TI "Eating Habit" OR AB "Eating Habit") OR (TI "habit eating" OR AB "habit eating") OR (TI "Dietary Habits" OR AB "Dietary Habits") OR (TI "Dietary Habit" OR AB "Dietary Habit") OR (TI "habit dietary" OR AB "habit dietary") OR (TI "Diet Habits" OR AB "Diet Habits") OR (TI "Diet Habit" OR AB "Diet Habit") OR (TI "habit diet" OR AB "habit diet") OR (TI "habits diet" OR AB "habits diet") OR (TI "dietary pattern" OR AB "dietary pattern") OR (TI "eating pattern" OR AB "eating pattern") OR (TI "food pattern" OR AB "food pattern"))
	AND
	(MH "meta analysis" OR MH "systematic review" OR MH "Technology, Medical/EV" OR PT "systematic review" OR PT "meta analysis" OR (((TI systematic* OR AB

	systematic*) N3 ((TI review* OR AB review*) OR (TI overview* OR AB overview*))) OR ((TI methodologic* OR AB methodologic*) N3 ((TI review* OR AB review*) OR (TI overview* OR AB overview*)))) OR (((TI quantitative OR AB quantitative) N3 ((TI review* OR AB review*) OR (TI overview* OR AB overview*) OR (TI synthes* OR AB synthes*))) OR ((TI research OR AB research) N3 ((TI integrati* OR AB integrati*) OR (TI overview* OR AB overview*)))) OR (((TI integrative OR AB integrative) N3 ((TI review* OR AB review*) OR (TI overview* OR AB overview*))) OR ((TI collaborative OR AB collaborative) N3 ((TI review* OR AB review*) OR (TI overview* OR AB overview*))) OR ((TI pool* OR AB pool*) N3 (TI analy* OR AB analy*))) OR ((TI "data synthes*" OR AB "data synthes*") OR (TI "data extraction*" OR AB "data extraction*") OR (TI "data abstraction*" OR AB "data abstraction*")) OR ((TI handsearch* OR AB handsearch*) OR (TI "hand search*" OR AB "hand search*")) OR ((TI "mantel haenszel" OR AB "mantel haenszel") OR (TI peto OR AB peto) OR (TI "der simonian" OR AB "der simonian") OR (TI dersimonian OR AB dersimonian) OR (TI "fixed effect*" OR AB "fixed effect*") OR (TI "latin square*" OR AB "latin square*")) OR ((TI "met analy*" OR AB "met analy*") OR (TI metanaly* OR AB metanaly*) OR (TI "technology assessment*" OR AB "technology assessment*") OR (TI HTA OR AB HTA) OR (TI HTAs OR AB HTAs) OR (TI "technology overview*" OR AB "technology overview*") OR (TI "technology appraisal*" OR AB "technology appraisal*")) OR ((TI "meta regression*" OR AB "meta regression*") OR (TI metaregression* OR AB metaregression*)) OR (MW meta-analy* OR MW metaanaly* OR MW "systematic review*" OR MW "biomedical technology assessment*" OR MW "bio-medical technology assessment*") OR ((TI medline OR AB medline OR MW medline) OR (TI cochrane OR AB cochrane OR MW cochrane) OR (TI pubmed OR AB pubmed OR MW pubmed) OR (TI medlars OR AB medlars OR MW medlars) OR (TI embase OR AB embase OR MW embase) OR (TI cinahl OR AB cinahl OR MW cinahl)) OR (SO Cochrane OR SO health technology assessment OR SO evidence report) OR ((TI comparative OR AB comparative) N3 ((TI efficacy OR AB efficacy) OR (TI effectiveness OR AB effectiveness))) OR ((TI "outcomes research" OR AB "outcomes research") OR (TI "relative effectiveness" OR AB "relative effectiveness")) OR (((TI indirect OR AB indirect) OR (TI "indirect treatment" OR AB "indirect treatment") OR (TI mixed-treatment OR AB mixed-treatment) OR (TI bayesian OR AB bayesian)) N3 (TI comparison* OR AB comparison*)) OR ((TI multi* OR AB multi*) N3 (TI treatment OR AB treatment) N3 (TI comparison* OR AB comparison*)) OR ((TI mixed OR AB mixed) N3 (TI treatment OR AB treatment) N3 ((TI meta-analy* OR AB meta-analy*) OR (TI metaanaly* OR AB metaanaly*))) OR (TI "umbrella review*" OR AB "umbrella review*") OR ((TI multi* OR AB multi*) N2 (TI paramet* OR AB paramet*) N2 (TI evidence OR AB evidence) N2 (TI synthesis OR AB synthesis)) OR ((TI multiparamet* OR AB multiparamet*) N2 (TI evidence OR AB evidence) N2 (TI synthesis OR AB synthesis)) OR ((TI multi-paramet* OR AB multi-paramet*) N2 (TI evidence OR AB evidence) N2 (TI synthesis OR AB synthesis))
Web of Science	TI=(("diet, vegetarian" OR "diet vegetarian" OR "diets vegetarian" OR "Vegetarian Diets" OR "Vegetarian Diet" OR "lacto vegetarian diet" OR "lacto vegetarian diet" OR "Lacto-Vegetarian Diets" OR "plant based diet" OR "diets plant based" OR "plant based diet" OR "Plant-Based Diets" OR "diet plant based" OR "diet plant based" OR "plant based nutrition" OR "nutrition plant based" OR "plant based nutrition" OR "lacto ovo vegetarian diet" OR "diet lacto ovo vegetarian" OR "diets lacto ovo vegetarian" OR "lacto ovo vegetarian diet" OR "Lacto-Ovo Vegetarian Diets" OR Vegetarianism OR Vegetarians OR Vegetarian OR Vegetarians OR Vegetarian OR "lacto ovo vegetarians" OR "lacto ovo vegetarians" OR "Lacto-Ovo Vegetarian" OR "vegetarian lacto ovo" OR "vegetarians lacto ovo" OR "ovo lacto vegetarians" OR "ovo lacto vegetarians" OR

	"Ovo-Lacto Vegetarian" OR "diet, vegan" OR "diet vegan" OR "diets vegan" OR "Vegan Diets" OR "Vegan Diet" OR Veganism OR vegans OR vegans OR vegan OR "seventh day adventists" OR "seventh day adventists" OR "Seventh-Day Adventist" OR "diet, western" OR "diet western" OR "diet, mediterranean" OR "mediterranean diet" OR "mediterranean diet" OR "Feeding Behavior" OR "Feeding Behavior" OR "behavior feeding" OR "Feeding Behaviors" OR "Eating Behavior" OR "behavior eating" OR "Eating Behaviors" OR "feeding related behavior" OR "feeding related behavior" OR "Feeding-Related Behaviors" OR "Feeding Patterns" OR "Feeding Pattern" OR "pattern feeding" OR "Food Habits" OR "Food Habit" OR "habit food" OR "Eating Habits" OR "Eating Habit" OR "habit eating" OR "Dietary Habits" OR "Dietary Habit" OR "habit dietary" OR "Diet Habits" OR "Diet Habit" OR "habit diet" OR "habits diet" OR "dietary pattern" OR "eating pattern" OR "food pattern"))
	AND
	TI=(("myocardial ischemia" OR "myocardial ischaemia" OR "ischemia myocardial" OR "ischemias myocardial" OR "Myocardial Ischemias" OR "Ischemic Heart Disease" OR "heart disease ischemic" OR "disease ischemic heart" OR "diseases ischemic heart" OR "heart diseases ischemic" OR "Ischemic Heart Diseases" OR "coronary disease" OR "coronary disease" OR "Coronary Diseases" OR "disease coronary" OR "diseases coronary" OR "Coronary Heart Disease" OR "Coronary Heart Diseases" OR "disease coronary heart" OR "diseases coronary heart" OR "heart disease coronary" OR "heart diseases coronary" OR "coronary artery disease" OR "coronary artery disease" OR "artery disease coronary" OR "artery diseases coronary" OR "Coronary Artery Diseases" OR "Left Main Coronary Artery Disease" OR "Left Main Disease" OR "Left Main Diseases" OR "Left Main Coronary Disease" OR "Coronary Arteriosclerosis" OR "atherosclerosis coronary" OR "Coronary Atherosclerosis" OR "arteriosclerosis coronary" OR "Coronary Heart Disease" OR "Myocardial Infarction" OR "Myocardial Infarction" OR "infarction myocardial" OR "infarctions myocardial" OR "Myocardial Infarctions" OR "Cardiovascular Stroke" OR "stroke cardiovascular" OR "strokes cardiovascular" OR "Myocardial Infarct" OR "infarct myocardial" OR "infarcts myocardial" OR "Myocardial Infarcts" OR "Heart Attack" OR "Heart Attacks" OR "acute coronary syndrome" OR "angina pectoris" OR "acute coronary syndrome" OR "angina pectoris" OR "Heart Arrest" OR "Heart Arrest" OR "arrest heart" OR "Cardiac Arrest" OR "arrest cardiac" OR Asystole OR Asystoles OR "Cardiopulmonary Arrest" OR "arrest cardiopulmonary" OR "death, sudden, cardiac" OR "death sudden cardiac" OR "Sudden Cardiac Death" OR "cardiac death sudden" OR "death sudden cardiac" OR "Cardiac Sudden Death" OR "death cardiac sudden" OR "sudden death cardiac" OR "Sudden Cardiac Arrest" OR "arrest sudden cardiac" OR "cardiac arrest sudden" OR "cardiovascular mortality" OR "cardiovascular death" OR stroke OR "Cerebrovascular Accident" OR Strokes OR "Cerebrovascular Accident" OR "Cerebrovascular Accidents" OR "cva cerebrovascular accident" OR "Cerebrovascular Apoplexy" OR "apoplexy cerebrovascular" OR "vascular accident brain" OR "Brain Vascular Accident" OR "Brain Vascular Accidents" OR "Cerebrovascular Stroke" OR "Cerebrovascular Strokes" OR "stroke cerebrovascular" OR "strokes cerebrovascular" OR Apoplexy OR "Cerebral Stroke" OR "Cerebral Strokes" OR "stroke cerebral" OR "strokes cerebral" OR "stroke acute" OR "Acute Stroke" OR "Acute Strokes" OR "strokes acute" OR "cerebrovascular accident acute" OR "Acute Cerebrovascular Accident" OR "Acute Cerebrovascular Accidents" OR "cerebrovascular accidents acute" OR "brain attack" OR "brain infarction" OR "brain infarction" OR "Brain Infarctions" OR "infarction brain" OR "infarctions brain" OR "Brain Infarct" OR "Brain Infarcts" OR "infarct brain" OR "infarcts brain" OR "Anterior Circulation Brain Infarction" OR "venous infarction brain" OR "Brain Venous Infarction" OR "venous

	infarctions brain" OR "Venous Brain Infarctions" OR "Anterior Cerebral Circulation Infarction" OR "brain infarction posterior circulation" OR "Posterior Circulation Brain Infarction" OR "Ischemic Stroke" OR "Ischemic Stroke" OR "carotid artery diseases" OR "carotid artery disease" OR "cerebral small vessel diseases" OR "cerebral small vessel disease" OR "intracranial artery disease" OR "cerebral arterial diseases" OR "intracranial arteriosclerosis" OR "arteriosclerosis intracranial" OR "Intracranial Atherosclerosis" OR "atherosclerosis intracranial" OR "Cerebral Arteriosclerosis" OR "arteriosclerosis cerebral" OR "Cerebral Atherosclerosis" OR "atherosclerosis cerebral" OR "intracranial embolism and thrombosis" OR "Cerebral Embolism and Thrombosis" OR "cerebral infarction" OR "cerebral ischemia" OR "cerebral ischaemia" OR "brain ischemia" OR "brain ischaemia"))
	AND filter “review”
Cochrane	vegetarian or vegan or "plant based diet" or veg*

Table S2 Credibility assessment of the results.
The class were adapted from [5–7].

Evidence	Class I (Convincing)	Class II (Highly suggestive)	Class III (Suggestive)	Class IV (Weak)	Non-significant
**P-value, meta-analysis**	$P < 10^{-6}$	$< 10^{-3}$	$P < 0.01$	$P \leqslant 0.05$	$P > 0.05$
**Number of cases**	n > 1000 (binary outcome) n > 20 000 (continuous outcome)	n > 1000 (binary outcome) n > 20 000 (continuous outcome)	n > 1000 (binary outcome) n > 20 000 (continuous outcome)	—	
**P-value, largest study**	< 0.05	< 0.05	—	—	
**95% CI, meta-analysis**	Exclude the null	—	—	—	
**Inconsistency score (I^2)**	< 50%	—	—	—	
**P-value, small study effects**	> 0.1	—	—	—	
**P-value, bias**	> 0.1	—	—	—	

Table S3 List of excluded articles in full-text screening with reason of exclusion

Author Year	DOI	Exclusion reason
Zyriax 2023	10.1016/j.maturitas.2022.09.003	Wrong outcomes
Zurbau 2020	10.1161/JAHA.120.017728	Wrong outcomes
StrÃ¶hle 2006	10.1007/s00508-006-0716-9	Wrong study design
Sofi 2012	10.4081/monaldi.2012.124	Wrong setting
Shirota 2022	10.3390/nu14102008	Wrong outcomes
Sherzai 2012	10.1111/j.1753-4887.2012.00490.x	Wrong intervention
Sanders 2014	10.3945/ajcn.113.071555	Wrong outcomes
Sahebkar 2023	10.2174/0929867331666230706102406	Wrong outcomes
Ravera 2016	10.3390/nu8060363	Wrong comparator
Pearson-Stuttard 2017	10.3945/ajcn.116.143925	Wrong outcomes
Ovesen 2005		Wrong study design
Oussalah 2020	10.1016/j.clnu.2020.02.037	Wrong study design
Mente 2009	10.1001/archinternmed.2009.38	Wrong outcomes
McEvoy 2012	10.1017/S1368980012000936	Wrong study design
Mangat 2009	10.3945/ajcn.2009.26736I	Wrong outcomes
Li 2015	10.1017/S000711451400289X	Wrong intervention
Kontogianni 2014	10.1016/j.maturitas.2014.06.014	Wrong intervention
Key 2003	10.1093/ajcn/78.3.533S	Wrong study design
Key 1998	10.1079/phn19980006	Wrong study design
Key 1999	10.1093/ajcn/70.3.516s	Wrong study design
Kaiser 2021	10.1093/jn/nxab037	Wrong outcomes
Hou 2015		Wrong intervention
Hartley 2013	10.1002/14651858.CD009874.pub2	Wrong outcomes
Ginter 2008		Wrong study design
Gan 2021	10.3390/nu13113952	Wrong intervention
Gan 2015	10.1016/j.ijcard.2015.01.077	Wrong intervention
Eilat-Adar 2010	10.1016/j.numecd.2010.01.011	Wrong outcomes
Dinu 2017	10.1080/10408398.2016.1138447	Wrong study design
DelRe 2022	10.1007/s11883-022-00981-4	Wrong intervention
Dauchet 2005	10.1212/01.wnl.0000180600.09719.53	Wrong intervention
Chhabra 2023	10.1177/02601060221122218	Wrong intervention
Charkviani 2022	10.3390/clinpract13010004	Wrong indication
Chareonrungrueangchai 2020	10.3390/nu12041088	Wrong study design
Bechthold 2019	10.1080/10408398.2017.1392288	Wrong outcomes
Aune 2019	10.1093/advances/nmz042	Wrong outcomes
Aune 2018	10.1093/ajcn/nqy097	Wrong outcomes
Agnoli 2023	10.1016/j.numecd.2023.04.005	Wrong study design
Babalola 2022	10.7759/cureus.29843	Wrong outcomes
Gardner 2001	10.1097/00019501-200111000-00005	Wrong study design
McCarty 2003	10.1016/S0306-9877(02)00241-4	Wrong outcomes
Mroskova 2021	10.32725/kont.2020.047	Wrong intervention
Roman 2019	10.1016/j.neurol.2019.08.005	Wrong outcomes
Luong 2022	10.1093/nutrit/nuab032	Wrong comparator
Satija 2017	10.1016/j.jacc.2017.05.047	Wrong study design

Garbett 2016	10.1016/j.nurpra.2016.04.013	Wrong indication
doRosario 2016	10.1093/nutrit/nuw012	Wrong study design
Key 2003	10.1093/ajcn/78.3.533s	Wrong study design
Lopes 2022	https://dx.doi.org/10.12688/f1000research.55220.2	Wrong study design
Sala-Vila 2022	https://dx.doi.org/10.1093/advances/nmac016	Wrong outcomes
Lin 2021	https://dx.doi.org/10.4103/tcmj.tcmj_168_20	Wrong outcomes
Amiot-Carlin 2022	https://dx.doi.org/10.1016/j.cnd.2022.07.002	Wrong outcomes
Chrysant 2022	https://dx.doi.org/10.1097/CRD.0000000000000392	Wrong indication
Doundoulakis 2021	https://dx.doi.org/10.1016/j.clnesp.2021.09.550	Wrong study design
deAquino 2020	https://dx.doi.org/10.1007/s13670-020-00335-5	Wrong intervention
Iguacel 2020	https://dx.doi.org/10.1007/s00198-020-05690-9	Wrong indication
Mukaneeva 2020	https://dx.doi.org/10.1177/2047487320935268	Wrong study design
Iacoviello 2018	https://dx.doi.org/10.1016/j.numecd.2017.12.010	Wrong study design
ArchundiaHerrera 2017	https://dx.doi.org/10.1007/s13679-017-0284-5	Wrong patient population
Panagiotakos 2016	https://dx.doi.org/10.2174/1570161114999160719104731	Wrong intervention
Bazal 2016	https://dx.doi.org/10.1093/eurheartj/ehw434	Wrong intervention
Jacques 2015	https://dx.doi.org/10.1017/S0007114515003141	Wrong outcomes
Sala-Vila 2015	https://dx.doi.org/10.1007/s11886-015-0583-y	Wrong outcomes
Pawlak 2015	https://dx.doi.org/10.1016/j.amepre.2015.02.009	Wrong intervention
Shen 2015	https://dx.doi.org/10.1146/annurev-nutr-011215-025104	Wrong intervention
Stradling 2014	http://dx.doi.org/10.2174/1871529X14666140701095426	Wrong outcomes
Casas 2014	http://dx.doi.org/10.2174/1871530314666140922153350	Wrong intervention
Fields 2014	https://dx.doi.org/10.1089/jwh.2014.4972	Wrong study design
McDermott 2014	https://dx.doi.org/10.1136/bmj.g4906	Wrong intervention
Messina 2014	https://dx.doi.org/10.3945/ajcn.113.071472	Wrong indication
Ekmekcioglu 2014		Wrong indication
Foroughi 2013		Wrong outcomes
FrancescoSofi 2013	https://dx.doi.org/10.1177/2047487314530052	Wrong outcomes
Chan 2013	https://dx.doi.org/10.1159/000354245	Wrong intervention
Singh 2012	https://dx.doi.org/10.2174/187639600120501009 0	Wrong study design
Hankey 2012	https://dx.doi.org/10.1016/S1474-4422%2811%2970265-4	Wrong intervention
Hill 2011	https://dx.doi.org/10.1007/s12170-011-0188-y	Wrong outcomes
Rabast 2008		Wrong study design
Mead 2006	https://dx.doi.org/10.1111/j.1365-277X.2006.00726.x	Wrong outcomes
Miettinen 2006	http://dx.doi.org/10.5414/CPP44247	Wrong intervention
Lock 2005		Wrong study design
Woodside 2005	https://dx.doi.org/10.1079/PNS2005464	Wrong outcomes
Singh 2003	https://dx.doi.org/10.1093/ajcn/78.3.526s	Wrong intervention

Dagnelie 2003		Wrong study design
Knight 1996		Wrong outcomes
Nestle 1995	http://dx.doi.org/10.1093/ajcn/61.6.1313S	Wrong intervention
An 2022	10.1016/j.jacc.2022.09.048	Wrong study design
Li 2022	10.3389/fnut.2022.963471	Wrong intervention
Li 2021	10.1016/j.foodchem.2021.130145	Wrong study design
Vernooij 2019	10.7326/M19-1583	Wrong intervention
Mann 2017	10.1016/B978-0-12-803968-7.00023-X	Wrong study design
Onvani 2017	10.1111/jhn.12415	Wrong outcomes
Zhang 2015	10.3390/nu7085300	Wrong intervention
RistiÄ‡-MediÄ‡ 2014		Wrong outcomes
Berciano 2014	10.1016/j.recesp.2014.05.003	Wrong study design
Singh 2014		Wrong study design
RodrÃguez-Monforte 2013	10.1017/S0007114515003177	Wrong intervention
Long 2010	10.1111/j.1741-6787.2009.00173.x	Wrong outcomes
Zeghichi-Hamri 2010	10.1016/j.nutres.2010.10.010	Wrong outcomes
Pino 2009		Wrong study design
Nestel 2005		Wrong outcomes
Spence 2003		Wrong outcomes
Holst 2002		Wrong outcomes

Table S4 Characteristics of included systematic reviews and meta-analyses.

**Author, year**	**Study design of included studies**	**Diet**	**Outcome**	**N studies**	**N participants**	**Dietary assessment method**	**Effect size**	**RoB tool**	**RoB**
Dinu et al. [8]	cohort; cross-sectional	Vegetarians	CVD	4	47757	Questionnaire	RR	NOS	12 Moderate and 5 Low
			IHD	6	65058				
Dybvik et al. [9]	cohort	Vegetarians	CVD	8	621282	FFQ	RR	ROBINS-I	For CVD and IHD: 3 studies are moderate RoB; 5 studies are serious RoB; For stroke: 7 studies are moderate RoB; 5 studies are serious RoB;
			IHD	8	621282				
			Stroke	12	770867				
Glenn et al. [10]	cohort	Vegetarians	CHD	1	44561	FFQ at baseline	RR	NOS	Only one cohort showed evidence of serious risk of bias
			CVD mortality	6	144247				
			CHD mortality	8	197737				
			Stroke	5	122525				
Huang et al. [11]	cohort	Vegetarians	IHD	6	124706	FFQ at baseline	RR	NOS	Begg's funnel plot and Begg's test showed a slight significant publication bias in ischemic heart disease and circulatory disease.
Jabri et al. [12]	cohort	Vegetarians	IHD mortality	7	125613	FFQ	RR	ROBINS-I	Most of the included studies had a moderate risk of bias
Jafari et al. [13]	cohort	Highest vs lowest vegetarians consumption	CVD mortality	12	168294	FFQ, Study dietary recall, Short general questionnaire	HR	NOS	Highly qualified studies received >= 7 stars
			CHD mortality	12	508561				

		Vegetarians vs nonvegetarians	CVD mortality	5	168294				
			CHD mortality	12	508561				
									The risk of bias was deemed to be moderate in five studies and low to moderate in two studies
Kwok et al. [14]	cohort	Vegetarian, seven days adventist	IHD	3	110723	FFQ, 24-hour dietary recalls, Food diaries, Self-reported elimination of animal products	RR		
		Vegetarian, non-seven days adventist	IHD	4	68246				
Lu et al. [15]	cohort	Vegetarians	Stroke	7	657433	FFQ	HR	NOS	
		Vegetarians	Ischemic Stroke	7	657433				
Quek et al. [16]	cohort	Plant-based dietary patterns	CVD mortality	13	410085	FFQs	HR	NOS	10 studies low risk of bias and 3 studies moderate risk of bias
			CVD	13	410085				
			stroke	13	410085				
		PDI, overall: highest vs lowest quintile	CVD mortality	13	410085				
			CVD	13	410085				
			CVD mortality	13	410085				

	CVD	13	410085
PDI, unhealthful: highest vs lowest quintile	CVD mortality	13	410085
	CVD	13	410085
Vegetarians	CVD mortality	13	410085
	CVD	13	410085
	stroke	13	410085

Abbreviations: CVD, cardiovascular disease; FFQ, food frequency questionnaire; IHD, Ischemic heart disease; HR, hazard ratio; NOS, Newcastle-Ottawa scale; PDI, Plant-based dietary Index; RR, risk ratio.

*1 study rely exclusively on self-identification of vegetarian status

Table S5 Credibility assessment of the results grouping by author and outcome of the meta-analysis included in the umbrella review.

Meta-analysis	Outcome	Sample Size	p-value significance	PI significance	Heterogeneity	Small study effect	Largest study significance	**Class***
Dinu 2016 [8]	CVD mortality	>1000	>0.05	null included	substantial	ns	Not null	ns
	IHD mortality	>1000	>0.05	null included	substantial	ns	Not null	ns
	Cerebrovascular disease	>1000	<0.05	null included	considerable	ns	Null	IV
Dybvik 2023 [9]	CVD incidence	>1000	<0.001	< 3 studies	only 1 study	< 3 studies	Not null	II
	CVD mortality	>1000	>0.05	< 3 studies	only 1 study	< 3 studies	Null	ns
	IHD incidence	>1000	<0.01	null included	considerable	ns	Not null	IV
	IHD mortality	>1000	<0.01	null included	substantial	ns	Not null	IV
	Stroke incidence	>1000	<0.001	< 3 studies	only 1 study	< 3 studies	Not null	III
	Stroke mortality	>1000	<0.05	null included	low	ns	Not null	IV
Glenn 2019 [10]	CVD mortality	>1000	>0.05	null included	substantial	ns	Not null	ns
	IHD incidence	>1000	<0.001	< 3 studies	only 1 study	< 3 studies	Not null	II
	IHD mortality	>1000	<0.05	null included	considerable	ns	Not null	IV
	Stroke mortality	>1000	>0.05	null included	considerable	ns	Not null	ns
Huang 2012 [11]	CVD mortality	>1000	>0.05	< 3 studies	substantial	< 3 studies	Not null	ns
	IHD mortality	>1000	<0.001	null included	considerable	ns	Not null	III

Meta-analysis	Outcome	Sample Size	p-value significance	PI significance	Heterogeneity	Small study effect	Largest study significance	**Class***
Jabri 2021 [12]	Stroke mortality	>1000	<0.05	null included	considerable	sig.	Not null	IV
	CVD mortality	>1000	<0.001	< 3 studies	only 1 study	< 3 studies	Not null	IV
	IHD mortality	>1000	<0.001	null included	moderate	ns	Not null	III
	Stroke mortality	>1000	>0.05	null included	considerable	ns	Not null	ns
Jafari 2022 [13]	CVD mortality	>1000	>0.05	null included	substantial	ns	Not null	ns
	IHD mortality	>1000	<0.001	null included	considerable	ns	Not null	III
	Stroke mortality	>1000	<0.05	null included	moderate	ns	Not null	IV
Kwok 2014 [14]	CVD mortality	>1000	>0.05	null included	substantial	ns	Not null	ns
	IHD mortality	>1000	<0.05	null included	substantial	ns	Not null	IV
	Stroke mortality	>1000	>0.05	null included	substantial	ns	Not null	ns
Lu 2021 [15]	Ischemic Stroke	>1000	<0.05	null included	moderate	ns	Not null	IV
Quek 2021 [16]	CVD mortality	>1000	<0.001	< 3 studies	only 1 study	< 3 studies	Not null	III
	Ischemic Stroke	>1000	<0.05	< 3 studies	moderate	< 3 studies	Not null	IV

Table S6 Credibility assessment of the results grouping by outcome of the meta-analysis included in the umbrella review.

Outcome	Sample Size	p-value significance	PI significance	Heterogeneity	Small study effect	Largest study significance
Cerebrovascular disease	>1000	<0.05	Null included	considerable	ns	Not null
CVD incidence	>1000	<0.001	< 3 studies	only 1 study	< 3 studies	Not null
CVD mortality	>1000	>0.05	Null included	substantial	ns	Null
IHD incidence	>1000	>0.06	Null included	substantial	ns	Not null
IHD mortality	>1000	<0.001	Null included	substantial	ns	Null
Ischemic Stroke	>1000	<0.05	Null included	considerable	ns	Not null
Stroke incidence	>1000	<0.001	< 3 studies	only 1 study	< 3 studies	Not null
Stroke mortality	>1000	>0.05	Null included	substantial	ns	Not null

Table S7 AMSTAR-2 scoring of the meta-analysis included in the umbrella systematic review

Author, Year	Q1	Q2*	Q3	Q4*	Q5	Q6	Q7*	Q8	Q9*	Q 10	Q 11*	Q12	Q13*	Q14	Q15*	Q16	AMSTAR-2
Dinu 2016	1	1	1	1	0	1	1	0	1	0	1	1	0	1	1	1	Low
Dybvik 2023	1	1	1	1	1	1	1	1	1	1	1	1	1	1	1	1	High
Glenn 2019	1	1	1	1	0	1	1	1	0	1	1	0	0	1	0	1	Critically low
Huang 2012	1	0	0	1	0	1	1	0	0	0	1	0	0	1	1	1	Critically low
Jabri 2021	1	1	1	1	1	1	1	1	0	0	1	0	0	1	0	1	Critically low
Jafari 2022	1		1	1	1	1	0	1	0	0	0	0	0	1	1	1	Critically low
Kwok 2014	1	1	1	1	1	1	0	1	0	0	1	1	1	1	1	1	Critically low
Liu 2022	1	1	1	1	0	1	1	0	1	0	1	0	0	0	1	1	Low
Lu 2021	1	1	1	1	1	1	0	0	1	1	1	1	1	0	1	1	Moderate
Quek 2021	1	1	1	1	1	1	1	0	1	0	1	1		1	1	1	Moderate

*Critical items items.

Q1. Did the research questions and inclusion criteria for the review include the components of PICO?
Q2. Did the report of the review contain an explicit statement that the review methods were established prior to the conduct of the review and did the report justify any significant deviations from the protocol?
Q3. Did the review authors explain their selection of the study designs for inclusion in the review?
Q4. Did the review authors use a comprehensive literature search strategy?
Q5. Did the review authors perform study selection in duplicate?
Q6. Did the review authors perform data extraction in duplicate?
Q7. Did the review authors provide a list of excluded studies and justify the exclusions?
Q8. Did the review authors describe the included studies in adequate detail?
Q9. Did the review authors use a satisfactory technique for assessing the risk of bias (RoB) in individual studies that were included in the review?
Q10. Did the review authors report on the sources of funding for the studies included in the review?
Q11. If meta-analysis was performed did the review authors use appropriate methods for statistical combination of results?
Q12. If meta-analysis was performed, did the review authors assess the potential impact of RoB in individual studies on the results of the meta-analysis or other evidence synthesis?
Q13. Did the review authors account for RoB in individual studies when interpreting/ discussing the results of the review?
Q14. Did the review authors provide a satisfactory explanation for, and discussion of, any heterogeneity observed in the results of the review?
Q15. If they performed quantitative synthesis did the review authors carry out an adequate investigation of publication bias (small study bias) and discuss its likely impact on the results of the review?
Q16. Did the review authors report any potential sources of conflict of interest, including any funding they received for conducting the review?

S8 References

1. SR / MA / HTA / ITC - CINAHL Available online: https://searchfilters.cadth.ca/link/98.
2. SR / MA / HTA / ITC - MEDLINE, Embase, PsycInfo Available online: https://searchfilters.cadth.ca/link/33.
3. SR / MA / HTA / ITC - PubMed Available online: https://searchfilters.cadth.ca/link/99.
4. SR / MA / HTA / ITC - Scopus Available online: https://searchfilters.cadth.ca/link/105.
5. Bellou, V.; Belbasis, L.; Tzoulaki, I.; Evangelou, E.; Ioannidis, J.P.A. Environmental Risk Factors and Parkinson's Disease: An Umbrella Review of Meta-Analyses. *Parkinsonism Relat Disord* **2016**, *23*, 1–9, doi:10.1016/j.parkreldis.2015.12.008.
6. Dinu, M.; Pagliai, G.; Casini, A.; Sofi, F. Mediterranean Diet and Multiple Health Outcomes: An Umbrella Review of Meta-Analyses of Observational Studies and Randomised Trials. *Eur J Clin Nutr* **2018**, *72*, 30–43, doi:10.1038/ejcn.2017.58.
7. Oussalah, A.; Levy, J.; Berthezène, C.; Alpers, D.H.; Guéant, J.-L. Health Outcomes Associated with Vegetarian Diets: An Umbrella Review of Systematic Reviews and Meta-Analyses. *Clinical Nutrition* **2020**, *39*, 3283–3307, doi:10.1016/j.clnu.2020.02.037.
8. Dinu M.; Pagliai G.; Casini A.; Sofi F. Vegetarian, Vegan Diets and Multiple Health Outcomes: A Systematic Review with Meta-Analysis of Observational Studies. *European Heart Journal* **2016**, *37*, 549, doi:10.1093/eurheartj/ehw432.
9. Dybvik JS; Svendsen M; Aune D Vegetarian and Vegan Diets and the Risk of Cardiovascular Disease, Ischemic Heart Disease and Stroke: A Systematic Review and Meta-Analysis of Prospective Cohort Studies. *Eur J Nutr* **2023**, *62*, 51–69, doi:10.1007/s00394-022-02942-8.
10. Glenn AJ; Viguiliouk E; Seider M; Boucher BA; Khan TA; Blanco Mejia S; Jenkins DJA; Kahleová H; Rahelić D; Salas-Salvadó J; et al. Relation of Vegetarian Dietary Patterns With Major Cardiovascular Outcomes: A Systematic Review and Meta-Analysis of Prospective Cohort Studies. *Front Nutr* **2019**, *6*, 80, doi:10.3389/fnut.2019.00080.
11. Huang T; Yang B; Zheng J; Li G; Wahlqvist ML; Li D Cardiovascular Disease Mortality and Cancer Incidence in Vegetarians: A Meta-Analysis and Systematic Review. *Ann Nutr Metab* **2012**, *60*, 233–240, doi:10.1159/000337301.
12. Jabri A; Kumar A; Verghese E; Alameh A; Khan MS; Khan SU; Michos ED; Kapadia SR; Reed GW; Kalra A Meta-Analysis of Effect of Vegetarian Diet on Ischemic Heart Disease and All-Cause Mortality. *Am J Prev Cardiol* **2021**, *7*, 100182, doi:10.1016/j.ajpc.2021.100182.
13. Jafari S; Hezaveh E; Jalilpiran Y; Jayedi A; Wong A; Safaiyan A; Barzegar A Plant-Based Diets and Risk of Disease Mortality: A Systematic Review and Meta-Analysis of Cohort Studies. *Crit Rev Food Sci Nutr* **2022**, *62*, 7760–7772, doi:10.1080/10408398.2021.1918628.
14. Kwok CS; Umar S; Myint PK; Mamas MA; Loke YK Vegetarian Diet, Seventh Day Adventists and Risk of Cardiovascular Mortality: A Systematic Review and Meta-Analysis. *Int J Cardiol* **2014**, *176*, 680–686, doi:10.1016/j.ijcard.2014.07.080.
15. Lu JW; Yu LH; Tu YK; Cheng HY; Chen LY; Loh CH; Chen TL Risk of Incident Stroke among Vegetarians Compared to Nonvegetarians: A Systematic Review and Meta-Analysis of Prospective Cohort Studies. *Nutrients* **2021**, *13*, doi:10.3390/nu13093019.
16. Quek J; Lim G; Lim WH; Ng CH; So WZ; Toh J; Pan XH; Chin YH; Muthiah MD; Chan SP; et al. The Association of Plant-Based Diet With Cardiovascular Disease and Mortality: A Meta-Analysis and Systematic Review of Prospect Cohort Studies. *Front Cardiovasc Med* **2021**, *8*, 756810, doi:10.3389/fcvm.2021.756810.
